# A cluster RCT and process evaluation of an implementation optimisation intervention to promote parental engagement enrolment and attendance in a childhood obesity prevention programme: results of the Optimising Family Engagement in HENRY (OFTEN) trial

**DOI:** 10.1186/s13063-021-05757-w

**Published:** 2021-11-05

**Authors:** Maria Bryant, Wendy Burton, Michelle Collinson, Amanda Farrin, Jane Nixon, June Stevens, Kim Roberts, Robbie Foy, Harry Rutter, Bethan Copsey, Suzanne Hartley, Sandy Tubeuf, Julia Brown

**Affiliations:** 1 Department of Health Sciences and the Hull York Medical School, University of York, YO105DD, York, UK; 2 Clinical Trials Research Unit, Leeds Institute of Clinical Trials Research, University of Leeds, Leeds, LS2 9JT UK; 3 Departments of Nutrition and Epidemiology, Gillings School of Public Health, University of North Carolina, Chapel Hill, NC 27599 USA; 4 HENRY Head Office, 8 Elm Place, Old Witney Road, Eynsham, OX29 4BD UK; 5 Academic Unit of Primary Care, Institute of Health Sciences, University of Leeds, Leeds, LS2 9JT UK; 6 London School of Hygiene and Tropical Medicine, 15-17 Tavistock Place, London, WC1H 9SH UK; 7 Academic Unit of Health Economics, Leeds Institute of Health Sciences, University of Leeds, Leeds, LS2 9JT UK; 8 IRSS-IRES, Université catholique de Louvain, B-1348 Louvain, La-Neuve Belgium

**Keywords:** Community, Parent, Engagement, Enrolment, Attendance, Obesity

## Abstract

**Background:**

Poor and variable implementation of childhood obesity prevention programmes reduces their population impact and sustainability. We drew upon ethnographic work to develop a multi-level, theory-based implementation optimisation intervention. This intervention aimed to promote parental enrolment and attendance at HENRY (Health Exercise Nutrition for the Really Young), a UK community obesity prevention programme, by changing behaviours of children’s centre and local authority stakeholders.

**Methods:**

We evaluated the effectiveness of the implementation optimisation intervention on HENRY programme enrolment and attendance over a 12-month implementation period in a cluster randomised controlled trial. We randomised 20 local government authorities (with 126 children’s centres) to HENRY plus the implementation optimisation intervention or to HENRY alone. Primary outcomes were (1) the proportion of centres enrolling at least eight parents per programme and (2) the proportion of centres with a minimum of 75% of parents attending at least five of eight sessions per programme. Trial analyses adjusted for stratification factors (pre-randomisation implementation of HENRY, local authority size, deprivation) and allowed for cluster design. A parallel mixed-methods process evaluation used qualitative interviews and routine monitoring to explain trial results.

**Results:**

Neither primary outcome differed significantly between groups; 17.8% of intervention centres and 18.0% of control centres achieved the parent enrolment target (adjusted difference − 1.2%; *95% CI* − 19.5%, 17.1%); 17.1% of intervention centres and 13.9% of control centres achieved the attendance target (adjusted difference 1.2%; *95% CI* − 15.7%, 18.1%). Unexpectedly, the trial coincided with substantial national service restructuring, including centre closures and reduced funds. Some commissioning and management teams stopped or reduced delivery of both HENRY and the implementation optimisation intervention due to competing demands. Thus, at follow-up, HENRY programmes were delivered to approximately half the number of parents compared to baseline (*n* = 433 vs. 881).

**Conclusions:**

During a period in which services were reduced by external policies, this first definitive trial found no evidence of effectiveness for an implementation optimisation intervention promoting parent enrolment to and attendance at an obesity prevention programme.

**Trial registration:**

ClinicalTrials.govNCT02675699. Registered on 4 February 2016

**Supplementary Information:**

The online version contains supplementary material available at 10.1186/s13063-021-05757-w.

## Introduction

Effectiveness evaluations indicate that investments in the design and delivery of public health interventions such as obesity prevention programmes are often not realised [1–4]. Key explanations for disappointing outcomes often concern low and variable implementation of public health programmes, including failure to ensure that a sufficient proportion of the target population participates (reach) and problems with the extent to which participants receive and interact with programme components (dose) [5]. Moreover, poor levels of enrolment and attendance at group-delivered programmes substantially undermine group dynamics and hence further compromise effectiveness and threaten programme viability [6–8]. Within group-delivered obesity prevention programmes, poor parental reach and dose occurs in the context of health inequalities and, in some cases, safeguarding concerns [9–11]. Low levels of service engagement are associated with socioeconomic and cultural factors and may indicate vulnerability, particularly in single-parent families, those with social or financial deprivation or families from ethnic minority groups [12].

Ideally, all new interventions would be developed and evaluated with ‘downstream’ implementation considerations in mind, to enable their translation from research into practice settings. However, this consideration of factors that are important from an end user perspective is not always done. It is therefore argued that evidentiary research (early-phase evaluation and optimisation of programmes) undertaken prior to the conduct of a large-scale clinical trial allows implementation factors to be addressed in advance, reducing financial waste and preventing type II error [13]. In the case of childhood obesity prevention programmes, where there is a lack of evidence demonstrating an effect, evidentiary research is much needed so that we can focus evaluation resources on programmes that we know can be successfully implemented in the real world to ensure their viability.

We developed and evaluated an intervention to optimise the implementation of an existing pre-school obesity prevention group programme, HENRY (Health, Exercise, Nutrition for the Really Young), prior to assessing the feasibility of undertaking a randomised controlled trial of its effectiveness in work which has been previously been published [14, 15]. HENRY is an 8-week programme delivered to groups of parents of preschool children. It was developed in 2006 with joint funds from the United Kingdom Department of Health and the former Department of Children, Schools and Families (now the Department for Education). It is commissioned and delivered nationally by 30–40 local authorities providing more than 150 programmes each year. Since it started, it has been delivered to an estimated 24,500 families. It is delivered in community settings, often by staff in children’s centres [16]. HENRY uses a responsive approach to provide practical guidance and improve parenting skills aimed at enhancing family lifestyle and children’s centre environments [17]. Despite some indications of the success of HENRY from audit [16, 18] and qualitative evaluations [17, 19, 20], routine monitoring indicates that implementation targets are often not met. Children’s centres rarely recruit the target of eight parents per programme (average is six) and only 60% of parents on average attend at least five out of eight sessions, thereby limiting programme reach and dose. Thus, in order to optimise the implementation of HENRY prior to assessing its effectiveness, enrolment and attendance levels needed to be addressed.

We developed the HENRY implementation optimisation intervention to promote programme attendance and enrolment. Development was informed by a focused ethnographic exploration of barriers and levers to parental enrolment and attendance in children’s centres delivering HENRY [21]. This found that barriers to enrolment and attendance mainly occurred at the organisational level, whereby children’s centre practices influenced how HENRY was perceived and experienced by parents. In addition, the extent to which local authorities prioritised HENRY had knock-on effects on local implementation and buy-in. The HENRY implementation optimisation intervention therefore targeted multiple organisational levels to support stakeholders, including local authority commissioners, children’s centre managers and staff, to promote enrolment and attendance. Our interdisciplinary team drew upon evidence on effective methods for promoting enrolment and attendance, the ethnography study and collective experience to develop the intervention. This is detailed elsewhere [22] and summarised below.

The Optimising Family Engagement in HENRY (OFTEN) trial evaluated the effectiveness of the implementation optimisation intervention in promoting parental enrolment and attendance at HENRY. Recognising the complexities of evaluating an intervention targeting multiple stakeholders during a period of national changes to local authority and children’s centre funding, we also undertook a comprehensive process evaluation to understand the influence of such contextual factors on trial findings. This paper reports both the trial findings and a summary of process evaluation findings.

## Methods

The implementation trial methods have been reported previously [23] and are summarised below.

### Aim

To determine the effectiveness of the optimisation intervention applied to HENRY compared with standard HENRY in regard to increasing parent enrolment in HENRY programmes or reducing parent attrition within HENRY programmes

### Study design and participants

**Fig. 1:**
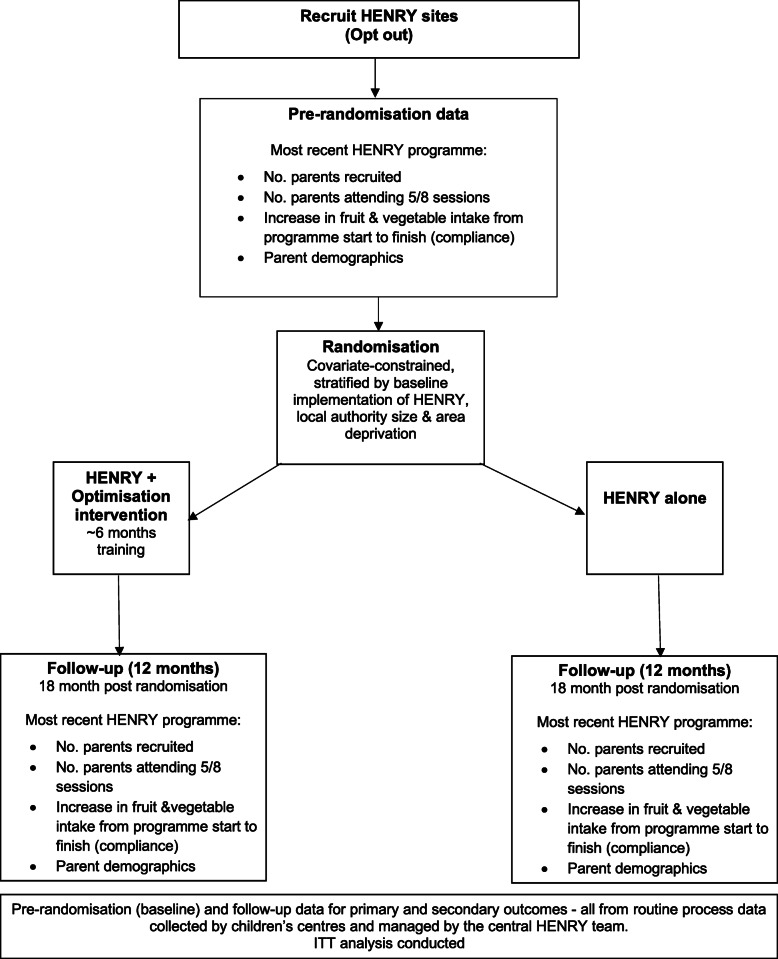
Study design

We conducted a two-arm, multi-centre, cluster randomised controlled trial (cRCT) across 20 local authorities in the UK. We compared the effects of the implementation optimisation intervention promoting parent enrolment and attendance at the HENRY programme to standard HENRY practice alone (Fig. 1). Although children’s centres deliver HENRY, due to the multi-level nature of the implementation optimisation intervention aiming to change behaviours of both children’s centre staff and local authority commissioners, we randomised at the level of local authorities (i.e. clusters) to reduce the likelihood of contamination between randomised groups. The School of Medicine Research Committee at the University of Leeds (MREC15-017) granted ethical approval for the study.

We recruited local authorities and children’s centres within them to the trial. Outcomes were obtained from routine data from the HENRY central office on enrolment, attendance and a proxy for parental compliance (changes in fruit and vegetable intake from the start to the end of the programme); thus, individual-level participant (i.e. parents) recruitment was not sought. For local authorities to meet inclusion criteria, they already had to be commissioning HENRY and consent for their centres to be involved in the research. Additionally, HENRY programmes had to be delivered by certified staff. Local authorities planning to decommission the HENRY intervention during the trial period were not eligible. Children’s centres were eligible if they provided data for the most recent HENRY programme delivered. Centres which participated in ethnographic work to develop the implementation optimisation intervention and those not planning to deliver any HENRY programmes during the trial period were excluded.

### Randomisation and masking

Local authorities were randomised in a 1:1 allocation ratio (HENRY + implementation optimisation intervention; HENRY alone) by a statistician at the Clinical Trials Research Unit (CTRU). An algorithm for covariate-constrained randomisation was used [24] to achieve a balanced allocation between the trial arms according to the following pre-randomisation factors: local authority level of parental engagement with HENRY (proportion of centres enrolling a minimum of eight parents per programme; proportion of centres retaining at least 75% of parents for a minimum of five out of eight sessions), proportion of centres delivering at least one HENRY programme in 2016, size of local authority (number of children’s centres participating with more or less than the median number of centres per local authority) and area deprivation (proportion of centres in the least and most deprived quintiles as ranked by the 2015 Index of Multiple Deprivation at the Lower Layer Super Output Area) [25].

Details of the optimisation were limited to a restricted number of central HENRY staff to avoid contamination (management team and named staff responsible for optimisation training). The central HENRY staff who were responsible for collating and transferring data to the CTRU were blinded to treatment allocation. It was not possible to blind allocation within intervention sites given the nature of the intervention. Families attending HENRY are routinely informed at enrolment that the programme uses data anonymously for research (website and privacy notice); they were not explicitly informed about the OFTEN trial or whether their local authority was assigned to the optimisation intervention.

### Procedures

Local authorities and their centres across the UK were identified through an existing database of HENRY delivery sites and invited to take part by direct invitation (posted or emailed by HENRY central office). An opt-out approach was used to promote efficiency and was approved due to the low-risk nature of the trial and low centre burden as outcomes were collected using routine data. Centres could decline participation in the study even if they were based within a consenting local authority. However, centres within areas where the local authority declined to take part in the trial were not eligible to participate. At the time of recruitment, 32 local authorities (317 children’s centres) in the UK ran the programme.

### HENRY alone (comparator)

Local authorities randomised to the HENRY alone arm continued to deliver HENRY programmes as per standard practice. HENRY is an 8-week programme delivered in children’s centres and aims to provide parents with skills, knowledge and confidence to support healthy behaviours among their preschool children. The theoretical underpinning combines evidenced-based models of behaviour change, including the Family Partnership Model, motivational interviewing and solution-focused support. Stage 1 training is designed to equip centre staff with the knowledge and skills to promote and provide healthy nutrition within early years settings and support parents to provide healthy family lifestyles and nutrition for their families. Stage 2 training supports practitioners to deliver the 8-week HENRY programme to families. This stage aims to build parents’ skills, knowledge and confidence to change old habits; provide healthier nutrition for their young children; and encourage healthier lifestyles [26, 27]. Programme content includes sessions on lifestyle and eating habits (e.g., family meals), balancing healthy meals and snacks, child-appropriate portion sizes, parenting, physical activity and emotional well-being.

### HENRY plus implementation optimisation intervention

The HENRY plus implementation optimisation arm delivered HENRY as standard, in addition to receiving components of the implementation optimisation intervention (see below). The Behaviour Change Wheel (BCW) framework [28] guided intervention development and was informed by the focused ethnography study [21], literature on promoting enrolment and attendance (e.g. [29, 30]), and experience and expertise of the implementation intervention development team. The development and final design of the intervention have been reported in full elsewhere [22]. The ethnography study [21] suggested that the starting point of an intervention to promote enrolment and attendance should begin at the organisational (local authority and children’s centre) levels. Local authority commissioner buy-in had a ‘spill-over’ effect on local implementation practices as it influenced their level of resource allocated to HENRY delivery. This, in turn, influenced how centre managers implemented HENRY; for example, the level to which HENRY was promoted (e.g. via posters, leaflets and displays) in centres and the number of staff that received training in HENRY. In centres not actively promoting HENRY, parents were not aware that programmes were running, limiting their opportunity to learn about and enrol on the programme. In centres where staff were not trained in the HENRY approach, their understanding of the programme was limited, further limiting information passed on to parents. Furthermore, centres seldom used simple strategies such as peer recruitment (i.e. word of mouth), yet it is known that parents are more likely to attend programmes if they are recommended by someone they trust [31, 32]. Facilitator skills also appeared important to promote enrolment and attendance, consistent with wider literature [33, 34]. Hence, the implementation optimisation intervention mainly aimed to change the behaviours of these multiple stakeholders—local authority commissioners, children’s centre managers and staff, HENRY facilitators and previous participants of HENRY.

Using BCW guidance, the intervention development team prioritised 11 target behaviours proposed to promote enrolment and attendance (Table 1). These included encouraging managers to increase the HENRY training provision for children’s centre staff and to initiate a peer recruitment initiative in their centres (whereby previous participants of HENRY would take an active role in recruiting friends and family). Overarching all centre-level target behaviours was the encouragement of local commissioners to support managers in their performance of centre-level behaviours by providing organisational, social and financial support. In order for the target behaviours to occur, the BCW framework offered guidance on intervention functions and behaviour change techniques to include in the intervention components. This process is reported separately [22]. The six intervention components that comprise the HENRY implementation optimisation intervention are detailed in Table 2 in line with guidance for intervention description reporting [41] and summarised here: (1) A local authority commissioner report designed to provide information to commissioners on how the HENRY programme benefits families that attend through the provision of parent-reported outcome data (e.g. changes in family eating behaviours and fruit and vegetable intake). This intervention component aimed to promote commissioner buy-in with HENRY and thus prioritise efforts to promote enrolment and attendance. (2) A commissioner overview leaflet designed to inform commissioners how suggested target behaviours were proposed to increase enrolment and attendance, with the aim of motivating them to support managers in their implementation of them. (3) A manager dashboard report designed to provide regular feedback to managers during follow-up on centre-level enrolment and attendance levels along with summarising parental behaviour change in order to persuade them to invest extra resources into HENRY engagement. (4) A half-day manager workshop introduced managers to the HENRY implementation optimisation intervention along with the target behaviours they were encouraged to perform, including goal setting and problem solving activities. (5) A HENRY facilitator refresher training session was designed to inform facilitators how they might enhance the participant experience to maintain attendance (e.g. allowing adequate time for group discussions to support the development of group bonds). In this session, facilitators were also asked to introduce the peer recruitment initiative to parents attending HENRY, and (6) existing HENRY promotional material (posters and leaflets) were revised to provide accurate information on what HENRY entailed and portray the holistic and inclusive nature of the programme.

**Table 1:** Recommended strategies for promoting parent engagement with HENRY

	Who	What	When	Rationale	Informed by	Proposed outcome
1.	Local authority commissioner	Support managers to perform target behaviours	Ongoing from the start of the intervention period	Local authority support for HENRY implementation optimisation intervention is likely to influence centre-level practices	Ethnography study findings and the implementation science literature (e.g. [35])	Manager performs target behaviours influencing enrolment and attendance
2.	Children’s centre manager	Hold ‘taster’ sessions prior to each HENRY programme where parents can attend an introductory session where the programme and format are explained	Prior to each delivered HENRY programme	Potential participants are more likely to engage if they have a greater understanding of what the programme entails	Experience of HENRY personnel, ethnography study finding (observation) and the literature (e.g. [36])	Parents have greater understanding of what HENRY is prior to enrolling influencing enrolment attendance
3.	Children’s centre manager	Increase HENRY training provision for centre staff	From the start of the intervention period	Some children’s centre staff lack knowledge of the HENRY programme and would benefit from training on the HENRY approach	Ethnography study (interviews and observation), experience of team members and the literature (e.g. [37])	Parents are provided with accurate information on what HENRY entails when approached to attend, influencing enrolment and attendance
4.	Children’s centre manager	[i] Hold HENRY programmes regularly and [ii] plan HENRY programmes far in advance	Ongoing from the start of the intervention	Some HENRY programmes are planned at short notice which hinders recruitment efforts	Ethnography study (informal conversations) and experience of the intervention development team	HENRY delivery is normalised and has greater visibility in centres influencing enrolment
5.	Children’s centre manager	Promote HENRY widely in centres using a range of methods	Ongoing from the start of the intervention	There is a general lack of awareness of HENRY among visiting parents	Ethnography study (observations, informal conversations and parent focus groups)	More parents are aware that HENRY programmes are running influencing enrolment
6.	Children’s centre manager	Allow a mix of referred and self-referred parents to enrol	Ongoing from the start of the intervention	Delivering programmes to a mix of parents (referred and self-referred) reduces barriers associated with stigma and improves group dynamics	Ethnography study (interviews and observations) and the literature (e.g. [38])	Staff approach more parents to attend and HENRY programmes are de-stigmatised influencing enrolment Group dynamics are improved influencing attendance
7.	Children’s centre manager and staff	Adopt a whole centre approach to HENRY; whereby [i] HENRY principles are adopted in other programmes and [ii] all staff are involved in the implementation of HENRY	Ongoing from the start of the intervention	Adopting a whole centre approach to HENRY implementation achieves better outcomes for engagement	Ethnography study (observations and informal conversations) and experience of the intervention development team	HENRY becomes more normalised and de-stigmatised in centres influencing enrolment Parents and staff have greater understanding of what programmes entail influencing enrolment and attendance
8.	Children’s centre staff	Promote HENRY accurately to dispel myths and negative perceptions	Ongoing from the start of the intervention	Misconceptions around what HENRY entails may deter people from engaging	Ethnography study (interviews, observations, focus group and informal interviews)	Parents understand what HENRY programme entails and are not put off by common misconceptions (e.g. that HENRY is a healthy eating programme) influencing enrolment and attendance
9.	HENRY facilitators	Ensure parents feel comfortable when attending the session by [i] considering characteristics of the parents before they attend and [ii] giving them enough time in sessions for group discussion	During all HENRY programmes	The skills of facilitators are known to influence engagement	Ethnography study (observation, focus groups and interviews) and the literature (e.g. [5, 39])	Parents feel comfortable attending (i.e. demonstrate confidence to engage) the session and form social bonds with other members of the group influencing attendance
10.	HENRY facilitators	Follow up on all parents that miss a session to encourage continued attendance	During all HENRY programmes	Participants feel valued if they are followed up after missing a session	Ethnography study (focus groups) and experience of the intervention development team	Parents are motivated to return to programme if a session is missed influencing attendance
11.	Previous HENRY participants	Encourage friends and family to engage with HENRY	Following HENRY programme attendance	Parents are more likely to attend a programme if they know someone that has attended before	Ethnography study (interviews and focus groups) and the literature (e.g [33, 40])	More parents are approached to enrol that are not already engaged with the centre and are more likely to sign up as they trust word of mouth recommendation influencing enrolment

**Table 2:** Components of the HENRY implementation optimisation intervention

Intervention component		Description	Recipient	Procedure	When
1	HENRY outcome report	Data provided to local authority commissioners on how HENRY benefits families that attend to motivate them to support managers in their performance of target behaviours Reported outcomes include enrolment and attendance and parent-reported behaviour change (e.g. changes to parenting efficacy and family eating behaviours)	Local authority commissioners	Data compiled by HENRY central team who produced and circulated the report to commissioners via email	Post-randomisation and after delivered HENRY programme (usually delivered in line with school terms)
	Commissioner overview leaflet	Leaflet provided to local authority commissioners to persuade them to support managers in their performance of target behaviours The leaflet includes a description of centre-level target behaviours and their proposed influence on enrolment and attendance	Local authority commissioners	Circulated by central HENRY team to commissioners via email	Post-randomisation
	Dashboard report	One page report circulated to managers to persuade them to perform target behaviours Report includes feedback on how many parents enrolled and attended the previous HENRY programme and the outcomes achieved by families that attended (e.g. changes to parenting efficacy and family eating behaviours)	Children’s centre managers	Data compiled by HENRY central team who circulated reports to HENRY coordinators via email who were responsible for circulating to centre managers	Post-randomisation and after each delivered HENRY programme
	Manager information workshop	Interactive half-day group workshops for managers delivered in each participating area to learn about the benefits of performing target behaviours along with goal setting and problem solving activities to persuade and enable them to perform them	Children’s centre managers	Local authority HENRY coordinators delivered the workshops at a local venue after receiving training from central HENRY office	Post-randomisation
	Facilitator refresher training	Interactive half-day group workshop delivered in each participating area for HENRY facilitators to receive training on how to perform target behaviours and receive information on the expected benefits of performing them. Facilitators are also instructed to introduce ‘peer’ recruitment to parents that attend HENRY to encourage them to recruit their friends and family	HENRY facilitators	Local authority HENRY coordinators delivered the workshops at a local venue after receiving training from the central HENRY office	Post-randomisation
	Revised promotional material (leaflets and posters)	Existing HENRY promotional material revised to more accurately portray what the HENRY programme entails, including a change to the tagline ‘Health, Exercise and Nutrition for the Really Young’ to ‘Healthy Family, Happy Home’ to better depict the holistic nature of the programme	Children’s centre staff and potential participants	Local authority HENRY coordinators distributed promotional material to centres to promote HENRY	Post-randomisation and throughout the follow-up period

HENRY national office was responsible for compiling and disseminating the commissioner report, dashboard report, overview leaflet and revised promotional material to participating local authorities and centres. Trainers from the HENRY national office provided training to local HENRY coordinators (who are responsible for coordinating HENRY activities within their area, typically with a background in public health delivery) on how to deliver manager and facilitator workshops within their areas. HENRY coordinators were responsible for coordinating and delivering manager workshops and facilitator refresher training sessions.

### Outcomes

As the intervention aimed to optimise the implementation of the HENRY programme prior to assessing its effectiveness, outcome measures were selected that reflected programme reach and dose received as these were previously identified implementation barriers. All local authorities which commission and deliver HENRY routinely provide process data to the central HENRY office for monitoring and quality assurance. These data are collected by the local HENRY delivery teams from parents at the start and end of each HENRY programme. After quality assurance checks, HENRY central office de-identified and shared these data with the OFTEN trial team. Except for data collected for the process evaluation, this trial only used these routine HENRY data in analysis of the primary and secondary outcomes.

Anonymised data that were transferred to the CTRU for the trial included enrolment and attendance data for programmes run pre-randomisation (baseline) and follow-up (programmes run for a 12-month period after training (6 months)) and anonymised parent-level data (child gender, age, ethnicity, the number and age of children under five in the home and questionnaire data (below)). As different families attend each HENRY programme, demographic characteristics differ between programmes delivered at baseline and follow-up.

#### Primary

The multiple primary outcomes were (i) the proportion of centres enrolling at least eight parents per programme and (ii) the proportion of centres with at least 75% of parents attending a minimum of five out of eight sessions per programme. The HENRY implementation optimisation intervention was to be considered to be effective if either the enrolment or retention goals were met. Justification of this approach was considered at length by the team and in discussion with the independent steering committee. Given that commissioners value both enrolment and attendance [42], it was agreed that improvements in either would be deemed effective (and subsequent adjustment was made to the analysis to account for multiplicity).

#### Secondary

The pre-specified secondary outcomes were:

1. Parental compliance to the HENRY programme (behaviour change) as measured via the proxy measure: proportion of parents reporting an increase of 0.5 in the daily frequency of consumption of fruits and vegetables by children per programme, measured by the modified and reduced Food Frequency questionnaire [43]
2. Proportion of children’s centres achieving all targets for enrolment, attendance and parent behaviour change
3. Longitudinal impact on enrolment and attrition assessed in children’s centres which provide data from more than one programme

### Sample size

Power calculations for a fixed sample size were conducted to examine the anticipated power for various intervention effects, in each of the primary outcomes and adjusting for multiplicity (see Additional Table 1 for scenarios). We assumed 25% of the 32 local authorities delivering HENRY would be ineligible or would opt out of the trial, leaving 24 local authorities (12 per arm). Based on data from previous HENRY programmes (2014), we assumed an average of 6 children’s centres per local authority, providing a total of 144 children’s centres (72 per arm), an intra-cluster correlation coefficient (*ICC*) between 0.05 and 0.1, a coefficient of variation in cluster size of 0.54 and the following estimates of the outcomes in the HENRY alone (standard practice) sites: 55% of centres will enrol at least eight parents per programme; 50% of centres will retain ≥75% of parents attending five of eight sessions.

Thus, with the anticipated number of centres (24 local authorities, 144 children’s centres), we expected to have at least 80% power to detect meaningful improvements in differences of 30% in the primary endpoints at the 5% significance level if the *ICC* was as high as 0.1 or at least 90% power to detect the same differences if the *ICC* was 0.05. Applying a Bonferroni correction to adjust for multiplicity arising from the analysis of multiple primary endpoints (alpha = 2.5%) would allow detection of a difference of 32% (slightly larger than the minimum meaningful improvement) in either of the primary endpoints if the *ICC* was 0.1 with at least 80% power or if the *ICC* was 0.05 with at least 90% power (see Additional Table 1 for scenarios).

### Process evaluation methods

A nested theory-driven process evaluation was undertaken alongside the trial. This approach uses an intervention’s ‘theory of change’ or logic model as the basis for evaluation by testing proposed assumptions that are built into the programme. The aim of this approach is to identify which assumptions do or do not hold to ensure the evaluation accurately reflects which programme activities are firmly connected to outcomes [44, 45]. As such, the process evaluation was designed to (1) assess whether the optimisation implementation intervention was delivered as planned (implementation), (2) explore whether change mechanisms proposed within the design of intervention components were enacted following receipt of the intervention, (3) measure performance of target behaviours and (4) explore the influence of contextual factors on the theory of change.

In this paper, we summarise the methods and results to report on the delivery of the implementation intervention, performance of target behaviours and key contextual factors which provide explanation of the trial result.

### Process evaluation methods; delivery of implementation intervention (dose delivered and fidelity of workshop delivery)

The level of dose received of each intervention component (commissioner report and leaflet, manager dashboard report and promotional material) was monitored per local authority using a distribution log (spreadsheet) to record which areas received which components and at which time points. These data were then summarised for each local authority. Assessment of whether manager and facilitator workshops were delivered in each local authority was assessed via email communication between the research team and local authority coordinators responsible for organising and delivering the workshops. Fidelity of workshop delivery was measured by using a workshop delivery checklist (completed by the workshop deliverer) to record whether content specified within the session plan (including behaviour change techniques) was delivered as planned. A researcher attended a number of workshops where permitted by the workshop deliverer and workshop attendees, who also completed a workshop delivery checklist in each workshop to validate self-report data.

### Process evaluation methods; performance of target behaviours

Due to the potential scale of the process evaluation component and the number of target behaviours, performance of behaviours was assessed at the commissioner and manager levels only, as these were the levels of the intervention that were proposed to have the biggest impact on parental enrolment and attendance [21]. Performance of commissioner-level behaviour change (providing support to managers in order for them to perform target behaviours) was explored via qualitative interview (as described below). At the manager level, process data routinely collated by HENRY central office was used to assess whether the following behaviours were performed in intervention and control centres: delivery of taster sessions, enrolling a mix of referred and self-referred participants, enrolling parents via peer support and increasing the number of HENRY programmes delivered from baseline to follow-up. These data were securely transferred to the CTRU at follow-up and handled and summarised by the trial statistician to describe the number of centres that performed each behaviour per trial arm. As routine data were not available to measure performance of all target behaviours at the manager level, a pre- and post-questionnaire was designed to measure whether the following practices changed from baseline to follow-up: the length of time HENRY programmes were planned in advance, the number of staff that attended HENRY training in the last 12 months, the way in which HENRY was promoted in the centre and incorporation of a whole centre approach of HENRY (e.g. the number and role of staff involved in HENRY implementation). The questionnaire was based on a self-assessment tool that is widely implemented in early year’s settings in the USA to assess health and well-being practices using Likert or numerical responses (e.g. in the past 12 months, HENRY programmes were usually planned approximately: 1 month in advance, 3–6 months in advance, 9–12 months in advance or longer than 12 months in advance) [46]. Questionnaire responses were compared from baseline to follow-up for each respondent. Where the numerical value increased from baseline to follow-up, it was assumed that the target behaviour had been performed by the children’s centre manager. Where the value decreased or stayed the same, it was assumed that the target behaviour had not been performed. The number of children’s centres performing the behaviour within each local authority was summarised per local authority and trial arm.

### Process evaluation methods; contextual factors

Interviews were held with commissioners and managers from local authorities in both arms of the trials to explore contextual factors that may have influenced and performance of target behaviours. A purposive sampling method was used to identify which commissioners and managers should be invited to take part in interviews. The aim of the sampling frame was to ensure representation of local authorities and children’s centres where participant engagement (HENRY enrolment and completion) had either increased, decreased or stayed the same from baseline to follow-up. All interviews were undertaken after the follow-up period to allow time for stakeholders time to reflect on their experiences during the trial. Written informed consent was received prior to all interviews taking place. All interviews were audio recorded using an encrypted secure device. Following transcription and checking of the data, the recordings were deleted. Interview data were analysed using inductive thematic analysis [47] whereby key words, phrases or sections of data were assigned an ‘initial code’ which reflected the content and nature of the data; for example, ‘funding constraints’, ‘staff capacity’ or ‘value placed on HENRY’. In the next stage, initial codes were reviewed to identify patterns between the codes and to group those that were similar, or discard those that were redundant or irrelevant. Codes were combined into themes that encapsulated overarching concepts. The themes were then reviewed against the transcripts to ensure they provided a true reflection of the data and that all participants’ perceptions and experiences were represented. A sub-section of data was second coded by a member of the research team before the final themes were agreed. Themes were then finalised and defined, and the data within them compared, contrasted and summarised.

### Statistical analysis

Analyses based on intention-to-treat (ITT) were conducted in SAS software version 9.4 (SAS Institute Inc. Cary NC) according to a pre-specified analysis plan. To adjust for two primary endpoints, a Bonferroni correction was applied and a two-sided significance level of 2.5% was used for each comparison, thereby preserving the family-wise error rate of 5%. All other endpoints were tested at the two-sided 5% significance level and no adjustments for multiple comparisons were made. Where centres ran more than one programme in the trial follow-up period, data from the last programmes delivered (most recent to analysis) in each centre were used in the primary analysis.

Due to the small number of clusters, a two-stage cluster-level analysis [40] of the primary outcomes was performed, adjusting for stratification factors (pre-randomisation levels of recruitment and attendance, proportion of centres delivering at least one HENRY programme in 2015, local authority size and area deprivation) [40]. Firstly, logistic regression models adjusted for stratification factors, but ignoring clustering of the data, were produced and residuals were summarised by cluster. A *t*-test was then performed on the cluster-level summaries of the covariate-adjusted residuals. If the distribution of the cluster-level summaries was skewed, the logarithm of the cluster-level summaries was used. Secondary outcomes were analysed using the same methods as the primary outcomes (with the exception of family eating behaviours and longitudinal impact on enrolment and attendance). Where applicable, secondary outcome models adjusted for the stratification factors, the change in the outcome at baseline (post-programme–pre-programme for the pre-randomisation programme) and the change in outcome at trial follow-up (i.e. for parent compliance, the model adjusted for the baseline change in parent-reported child intake of fruits and vegetables and parent-reported child intake of fruits and vegetables). *ICC*s were calculated using mixed effects models adjusted for the stratification factors. Missing item-level data was imputed for the self-efficacy measure using the half rule because this was the only continuous outcome measure where multiple items were summed to calculate a total score [48]. Missing data were not imputed for any other measures or for the primary outcomes [49]; if a children’s centre did not deliver a HENRY programme during the trial (post-randomisation), they were still included in the analysis, under ITT, and classified as not having met the enrolment or attendance target.

### Changes to methods after trial registration

A 6-month period for training of the implementation optimisation intervention was added prior to the trial 12-month trial intervention delivery period in which HENRY programmes were delivered at participating children’s centres (extending the follow-up to 18 months post-randomisation). In addition, our original protocol stated that we would conduct a full cost-benefit analysis of the optimisation intervention. However, subsequent null trial findings indicated that this was not appropriate. Instead, a discrete choice study [50] was conducted to consider, more widely, what delivery elements of obesity prevention programmes are most valued by commissioners [42]. The sample size within the published protocol did not allow for analysis of two primary endpoints and incorrectly included reference to a single composite endpoint. This has been updated both in the text and in Additional Table 1.

## Results

### Recruitment and participant flow

#### Local authorities

**Fig. 2:**
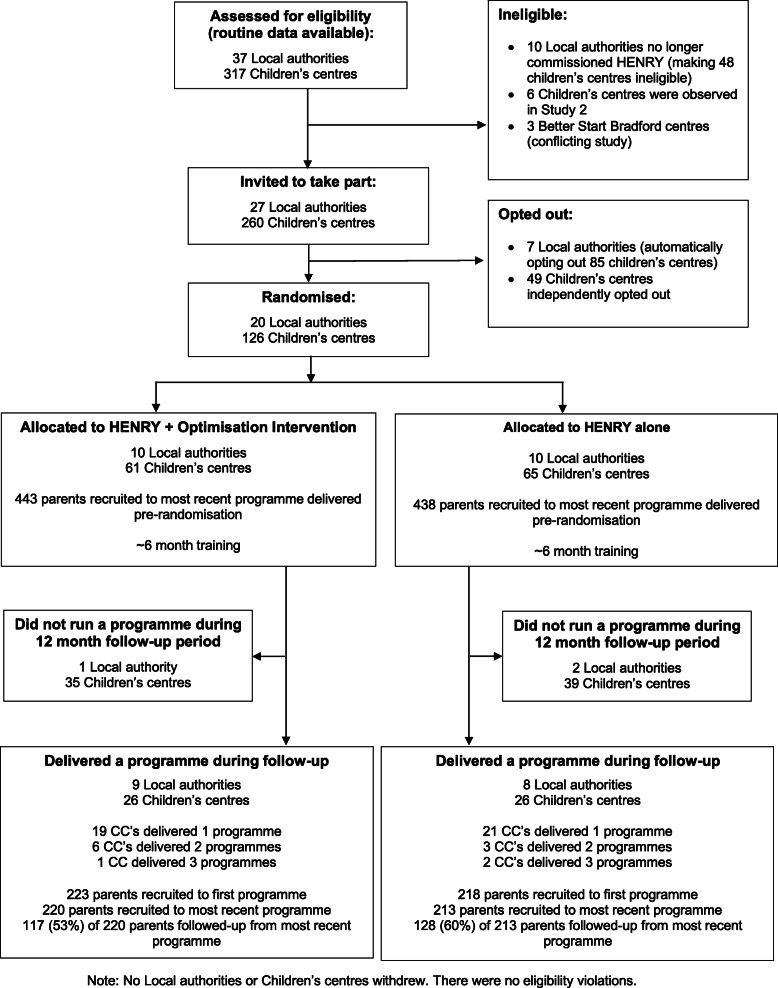
CONSORT flow diagram

Figure 2 shows the flow of local authorities, children’s centres and parents during the trial. Between 1 January 2016 and 30 March 2016, 37 local authorities, supporting 317 children’s centres, were screened for eligibility. Ten (27%) local authorities no longer commissioned HENRY and seven (19%) opted out. The remaining 20 (54%) local authorities (supporting 126 children’s centres) were recruited and randomised into either HENRY + optimisation intervention (*n* = 10) or HENRY alone (*n* = 10). Pre-randomisation characteristics for local authorities were well balanced between the arms (Table 3).

**Table 3:** Local authority pre-randomisation characteristics by arm

	HENRY alone (***n*** = 10)	HENRY + optimisation intervention (***n*** = 10)	Total (***n*** = 20)
Number of children’s centres	65	61	126
Proportion of children’s centres meeting the recruitment target of at least 8 parents per programme			
Mean (*SD*)	0.5 (0.3)	0.6 (0.3)	0.5 (0.3)
Proportion of children’s centres meeting the attendance target of at least 75% parents attending 5/8 sessions per programme			
Mean (*SD*)	0.5 (0.3)	0.5 (0.3)	0.5 (0.3)
Proportion of children’s centres running at least one HENRY programme in 2015			
Mean (*SD*)	0.7 (0.3)	0.8 (0.3)	0.7 (0.3)
Size of local authority			
Less than the median number of children’s centres per local authority	5 (50.0%)	5 (50.0%)	10 (50.0%)
More than the median number of children’s centres per local authority	5 (50.0%)	5 (50.0%)	10 (50.0%)
Proportion of children’s centres in the most deprived quintile			
Mean (*SD*)	0.6 (0.4)	0.6 (0.3)	0.6 (0.3)
Proportion of children’s centres in the least deprived quintile			
Mean (*SD*)	0.0 (0.1)	0.0 (0.1)	0.0 (0.1)

### Pre-randomisation data on HENRY programme attendees

Demographic characteristics for the 881 parents enrolled into HENRY programmes pre-randomisation were generally balanced by arm although some imbalances in ethnicity were evident (Table 4). There was a high volume of individual-level routine data missing although this was balanced between the arms: 24 children’s centres were unable to provide questionnaire data for their parents and for centres which provided data, not all data were available for some parents enrolled onto programmes. Reasons for missing questionnaire data included invalid for processing (*n* = 19), not returned to the central office (*n* = 3) and incorrect measures used (*n* = 2). Where parent questionnaire data was available, most parents were female, aged between 25 and 64 years and had heard about the HENRY programme via professional referral (e.g. children’s centre staff, health visitor, family support worker). Demographic characteristics for local authorities are presented in Additional Tables 2 to 6.

**Table 4:** Participant characteristics by arm

	Pre-randomisation			Follow-up		
	Centres subsequently allocated to HENRY alone (*n* = 438)	Centres subsequently allocated to HENRY + optimisation intervention (*n* = 443)	Total (*n* = 881)	Centres delivering HENRY alone (*n* = 213)	Centres delivering HENRY + optimisation intervention (*n* = 220)	Total (*n* = 433)
Parent gender						
Male	13 (3.0%)	9 (2.0%)	22 (2.5%)	8 (3.8%)	3 (1.4%)	11 (2.5%)
Female	219 (50.0%)	215 (48.5%)	434 (49.3%)	120 (56.3%)	112 (50.9%)	232 (53.6%)
Prefer not to say	4 (0.9%)	4 (0.9%)	8 (0.9%)	0 (0.0%)	2 (0.9%)	2 (0.5%)
Missing	202 (46.1%)	215 (48.5%)	417 (47.3%)	85 (39.9%)	103 (46.8%)	188 (43.4%)
Parent age						
18	0 (0.0%)	0 (0.0%)	0 (0.0%)	0 (0.0%)	0 (0.0%)	0 (0.0%)
18–25 yrs	46 (10.5%)	36 (8.1%)	82 (9.3%)	20 (9.4%)	16 (7.3%)	36 (8.3%)
25–64 yrs	185 (42.2%)	185 (41.8%)	370 (42.0%)	107 (50.2%)	99 (45.0%)	206 (47.6%)
65 yrs+	2 (0.5%)	2 (0.5%)	4 (0.5%)	0 (0.0%)	1 (0.5%)	1 (0.2%)
Prefer not to say	3 (0.7%)	5 (1.1%)	8 (0.9%)	1 (0.5%)	1 (0.5%)	2 (0.5%)
Missing	202 (46.1%)	215 (48.5%)	417 (47.3%)	85 (39.9%)	103 (46.8%)	188 (43.4%)
Number of children^a^	*n* = 232	*n* = 282	*n* = 514	*n* = 183	*n* = 169	*n* = 352
< 1 yrs^a^	65 (28.0%)	68 (24.1%)	133 (25.9%)	43 (23.5%)	34 (20.1%)	77 (21.9%)
1 yrs^a^	59 (25.4%)	71 (25.2%)	130 (25.3%)	41 (22.4%)	38 (22.5%)	79 (22.4%)
2 yrs^a^	34 (14.7%)	43 (15.2%)	77 (15.0%)	29 (15.8%)	31 (18.3%)	60 (17.0%)
3 yrs^a^	34 (14.7%)	36 (12.8%)	70 (13.6%)	28 (15.3%)	29 (17.2%)	57 (16.2%)
4 yrs^a^	27 (11.6%)	38 (13.5%)	65 (12.6%)	25 (13.7%)	15 (8.9%)	40 (11.4%)
5 yrs^a^	13 (5.6%)	26 (9.2%)	39 (7.6%)	17 (9.3%)	22 (13.0%)	39 (11.1%)
Ethnicity						
African	7 (1.6%)	15 (3.4%)	22 (2.5%)	8 (3.8%)	5 (2.3%)	13 (3.0%)
Arab	1 (0.2%)	0 (0.0%)	1 (0.1%)	0 (0.0%)	2 (0.9%)	2 (0.5%)
Bangladeshi	2 (0.5%)	9 (2.0%)	11 (1.2%)	2 (0.9%)	3 (1.4%)	5 (1.2%)
Black UK	5 (1.1%)	1 (0.2%)	6 (0.7%)	1 (0.5%)	1 (0.5%)	2 (0.5%)
Caribbean	1 (0.2%)	6 (1.4%)	7 (0.8%)	2 (0.9%)	2 (0.9%)	4 (0.9%)
Chinese	1 (0.2%)	2 (0.5%)	3 (0.3%)	2 (0.9%)	0 (0.0%)	2 (0.5%)
English/Scottish/Welsh/Northern Irish/UK	181 (41.3%)	146 (33.0%)	327 (37.1%)	75 (35.2%)	61 (27.7%)	136 (31.4%)
Gypsy or Irish traveller	0 (0.0%)	1 (0.2%)	1 (0.1%)	0 (0.0%)	0 (0.0%)	0 (0.0%)
Indian	6 (1.4%)	4 (0.9%)	10 (1.1%)	4 (1.9%)	1 (0.5%)	5 (1.2%)
Irish	0 (0.0%)	0 (0.0%)	0 (0.0%)	0 (0.0%)	1 (0.5%)	1 (0.2%)
Mixed ethnic background	2 (0.5%)	2 (0.5%)	4 (0.5%)	7 (3.3%)	2 (0.9%)	9 (2.1%)
Pakistani	3 (0.7%)	12 (2.7%)	15 (1.7%)	5 (2.3%)	11 (5.0%)	16 (3.7%)
Any other Asian background	5 (1.1%)	1 (0.2%)	6 (0.7%)	3 (1.4%)	1 (0.5%)	4 (0.9%)
Any other Black/African/Caribbean background	1 (0.2%)	1 (0.2%)	2 (0.2%)	2 (0.9%)	2 (0.9%)	4 (0.9%)
Any other White background	13 (3.0%)	13 (2.9%)	26 (3.0%)	0 (0.0%)	0 (0.0%)	0 (0.0%)
Any other ethnic group	3 (0.7%)	3 (0.7%)	6 (0.7%)	0 (0.0%)	9 (4.1%)	9 (2.1%)
Prefer not to say	5 (1.1%)	12 (2.7%)	17 (1.9%)	7 (3.3%)	5 (2.3%)	12 (2.8%)
Missing	202 (46.1%)	215 (48.5%)	417 (47.3%)	95 (44.6%)	114 (51.8%)	209 (48.3%)
How did parents hear about the HENRY programme?						
Family and friends	10 (2.3%)	4 (0.9%)	14 (1.6%)	9 (4.2%)	5 (2.3%)	14 (3.2%)
Leaflet	10 (2.3%)	15 (3.4%)	25 (2.8%)	14 (6.6%)	10 (4.5%)	24 (5.5%)
Poster	5 (1.1%)	2 (0.5%)	7 (0.8%)	7 (3.3%)	2 (0.9%)	9 (2.1%)
Professional	63 (14.4%)	46 (10.4%)	109 (12.4%)	89 (41.8%)	74 (33.6%)	163 (37.6%)
Website	2 (0.5%)	1 (0.2%)	3 (0.3%)	1 (0.5%)	0 (0.0%)	1 (0.2%)
Other	10 (2.3%)	18 (4.1%)	28 (3.2%)	8 (3.8%)	15 (6.8%)	23 (5.3%)
Missing	338 (77.2%)	357 (80.6%)	695 (78.9%)	85 (39.9%)	114 (51.8%)	199 (46.0%)

^a^Collected as the number of children of each age per parent, numbers reported therefore total more than the number of parents pre-randomisation/follow-up

^b^Routine data were missing for 417 parents pre-randomisation and 188 parents at follow-up

### Implementation optimisation intervention delivery

Outcomes were assessed during the delivery of HENRY programmes between 1 September 2016 and 30 August 2017. Fifty-two of 126 (41%) children’s centres (26 HENRY + optimisation; 26 HENRY alone) from seventeen (85%) local authorities delivered at least one HENRY programme. Of the remaining seventy-four children’s centres, 35 of 61 centres (57%) from one local authority in the HENRY + optimisation intervention group, and 39 of 65 children’s centres (60%) from two local authorities in the HENRY alone arm, did not deliver a HENRY programme.

Seventy-four centres did not deliver HENRY predominantly because local authorities scheduled a reduced number of programmes (for parents across the local authority to attend) rather than scheduling delivery in every centre (*n* = 25). Other reasons provided included the following: HENRY programmes on hold due to ‘major restructuring’ or ‘upheaval in centres’ (*n* = 17), HENRY being scaled down or de-commissioned (*n* = 7), lack of HENRY facilitators in post (*n* = 4), limited resources (*n* = 3) or centre closure (*n* = 3). Ten centres did not provide a reason and five centres cancelled HENRY programmes due to low uptake. No local authorities or children’s centres actively withdrew from the trial.

Participant characteristics were broadly similar to those observed pre-randomisation and similar quantities of missing data were observed overall; however, parents in the HENRY alone arm had less missing data compared to the HENRY + optimisation intervention (Table 4). Demographic characteristics for local authorities are presented in Additional Tables.

### Primary outcomes

Post-randomisation primary outcomes did not differ significantly between the groups: proportion of children’s centres enrolling at least eight parents per programme (adjusted risk difference = −1.2%, *95% CI* = −19.5%, 17.1%, *p* = 0.886) and proportion of children’s centres with at least 75% of parents attending 5/8 sessions per programme (adjusted risk difference = 1.2%, *95% CI* = −15.7%, 18.1%, *p* = 0.881) (Table 5).

**Table 5:** Primary outcomes: pre-randomisation proportions, outcome proportions and risk differences adjusted for stratification factors

	Pre-randomisation^a^ (%)	Unadjusted model estimates^b^			Adjusted model estimates^bc^		
		Outcome (%)	*RD* (*95% CI*)	*p*-value	*RD* (*95% CI*)	*p*-value	*ICC*
Primary outcome 1: enrolment							
HENRY alone (*n* = 10 local authorities)	50.0	18.0	−0.3 (−19.1, 18.6)	0.978	−1.2 (−19.5, 17.1)	0.886	0.136
HENRY + optimisation intervention (*n* = 10 local authorities)	60.0	17.8					
Primary outcome 2: attendance							
HENRY alone (*n* = 10 local authorities)	50.0	13.9	3.1 (−13.3, 19.6)	0.695	1.2 (−15.7, 18.1)	0.881	< 0.001
HENRY + optimisation intervention (*n* = 10 local authorities)	50.0	17.1					

^a^Calculation of outcomes used data provided for randomisation

^b^Calculation of outcomes used data from the most recently delivered HENRY programme during follow-up at 18 months post-randomisation

^c^Variables controlled for in the adjusted analyses were as follows: proportion of children’s centres recruiting at least 8 parents per programme at randomisation, proportion of children’s centres retaining at least 75% of parents for a minimum of 5/8 sessions per programme at randomisation, proportion of children’s centres running at least one HENRY programme in 2015, size of local authority and proportion of children’s centres in the least/most deprived quintile as ranked by the 2015 Index of Multiple Deprivation at the Lower Layer Super Output Area

*Abbreviations*: *RD* risk difference, *CI* confidence interval, *ICC* intra-cluster correlation coefficient

### Secondary outcomes

There was little evidence of any intervention effects for the secondary outcomes of change in fruit and vegetable intake (proxy to compliance) and the composite outcome including enrolment, attendance and compliance (Additional Tables 12 and 13). Missing data was substantial for parent-reported secondary outcomes; routinely collected questionnaire data was available for 245 (56%) parents pre/post-programme (60% HENRY alone vs. 53% HENRY + optimisation intervention) compared to 881 (100%) of parents pre-randomisation.

### Process evaluation

#### Delivery of implementation optimisation intervention components

Delivery of the HENRY implementation optimisation intervention components varied between local authorities and was delivered in full in just four out of the ten local authorities which hindered its ability to instigate behaviour change and hence promote parental engagement. The commissioner overview leaflet was delivered to all but one local authority but the commissioner outcome report was delivered at the appropriate time points in just three local authorities. Dashboard reports were not delivered at the appropriate time points in any of the local authorities. Manager workshops were delivered in the specified format in four out of ten local authorities. Workshop delivery checklists were received from four out of seven workshops reporting that two delivered 100% of the specified behaviour change techniques and one delivered 78% but one local authority delivered only 40%. Facilitator workshops were delivered in the specified format in five local authorities: delivery checklists were received from all workshops with all reporting that 100% of behaviour change techniques were delivered. Uptake of the re-branded promotional material was lower than expected, with just four local authorities using the materials.

#### Implementation optimisation intervention behaviour change

Target behaviours performed at the manager level were measured via routine data on the delivery of taster session, enrolling a mix of referred and self-referred parents, enrolling via peer recruitment and increasing the number of programme delivered per year. Data showed that some target behaviours were performed in both intervention and control centres (Table 6). With the exception of the delivery taster sessions, the number of centres performing target behaviours was similar between arms. As the numbers were small, no statistical analyses were performed. Analysis was not performed to assess behaviour change from pre-randomisation to follow-up, so it is possible that some centres in both arms were already using the strategies pre-randomisation. It was not possible to assess performance of the remaining target behaviours (planning programmes far in advance, provision of HENRY training, promoting HENRY using a variety of methods, and adopting a whole centre approach of HENRY) due to the poor return of questionnaire data. Potential relationships between adoption of the strategies and parent enrolment and completion outcomes were explored but there was no indication of a causal link.

**Table 6:** Number of centres performing target behaviours that were measurable using process data

	Control *N* (*n* = 26)	Intervention *N* (*n* = 26)	Total *N* (*n* = 52)
Delivery of taster session			
Yes	5	14	19
No	20	9	29
Missing	1	3	4
Total	26	26	52
Mix of referred and self-referred parents			
Yes	9	8	17
No	8	8	16
Missing	9	10	19
Total	26	26	52
Parents recruited via peer support			
Yes	4	5	17
No	21	16	16
Missing	1	5	19
Total	26	26	52
Increased number of HENRY programmes delivered			
Yes	7	7	14
No	19	19	38
Total	26	26	52

#### Contextual factors

Seventeen interviews were conducted between May and October 2018 with participants from intervention and control arms which explored contextual factors; seven from the HENRY alone arm (commissioners *n* = 3; manager/centre representative *n* = 4) and ten from the intervention arm (commissioners *n* = 3; manager/centre representative *n* = 7). Qualitative analysis of interview data highlighted three key contextual themes which provide explanation of the results: organisational change and reduced funding, parent engagement efforts outside of the study and the delivery of HENRY programmes. A summary of these findings is presented by theme.

##### Organisational change and reduced funding

The cuts to funding brought on by austerity measures led to many local authorities in England scaling back children’s centre services, resulting in reduced budgets, the amalgamations of centres and job losses [51, 52]. The cutbacks caused uncertainty among some managers around whether their centre would remain open, and the types of services that would be offered moving forward. This problem was described by some managers as overshadowing engagement with the study:

Looking at what was going on in the local authority at the time, it probably wasn’t the best time for us to be part of that study. Cos I know through kind of the end of 2015-2016 they were just starting to get rid of managers left, right and centre so unfortunately I don’t think HENRY was probably top of their radar if I’m completely honest. (HENRY + optimisation intervention manager)

Reduced capacity and funds were also reported by some managers as barriers to delivering the recommended engagement strategies such as taster sessions; therefore, in some centres, behaviour change did not occur that was proposed to promote engagement:

Yes taster sessions was something that we did talk about, but we just didn’t have capacity to do really. People just think “oh well it’s a taster session” but actually it’s getting ready for that session, doing the session, and looking at it afterwards and a lot of planning and preparation you know has to go into” (HENRY + optimisation intervention manager)

Furthermore, despite the aim of the intervention being to increase enrolment and completion to HENRY, some centres were not able to increase their programme capacity due to renewed financial constraints on the number of crèche places available to support parents attending HENRY. Although crèche limitations were identified during the intervention development work, an overarching aim of the intervention was to promote local authority and manager buy-in with HENRY to support engagement efforts:

If you want more people in then you have to provide the crèche staff […] that’s always been probably the most challenging aspect (HENRY + optimisation intervention manager)

##### Parental engagement efforts outside of the study

During the study, centres in both trial arms still sought to promote engagement with HENRY using initiatives outside of the trial. This was a consequence of undertaking pragmatic research in this setting, where despite taking part in a trial which aimed to test a specific set of engagement strategies, participating centres routinely continued to engage as many participants as possible to HENRY to ensure value for money and make the best use of resources. Therefore, centres from both trial arms tried out strategies of their own. For example, some managers and commissioners described how they undertook pre-home visits prior to HENRY programmes to promote engagement:

We’ve started home visits in the last couple of years, and it varies on who we’ve got coming on it but sometimes it is best to be able to go out and do a home visit prior to the course so you can see them in their own environment, and then other times we’ve tried doing like a coffee morning but we’ve found the home visits more successful than the coffee morning. (HENRY alone commissioner)

In addition, quantitative data on the uptake of optimisation strategies showed that some centres in the HENRY alone used engagement strategies that were part of the intervention optimisation e.g. taster sessions. This contamination may have been due to prior relationships with the HENRY central team or attendance at regional network meetings where the same strategies for promoting engagement may have been suggested either before or during the trial. Or through centres using the same strategies with similar programmes:

We do the taster session. That was from the children’s centres saying it worked with other parenting courses; like holding a pre session to like de-mystify it so the parents weren’t scared. (HENRY alone commissioner)

##### Delivery of HENRY programmes

As described above, a large proportion of centres from both trial arms did not deliver a programme during the trial due to reasons such as limited staff capacity. During interviews, managers also described how local authority scheduling influenced whether they delivered a programme in their centre:

We work as part of a cluster, we do one big cluster timetable […] and we alternate between a nurturing programme and HENRY, each site will do HENRY one term and then they’ll do a nurturing the next time (HENRY + optimisation intervention manager)

In addition, some managers perceived HENRY as being resource heavy in terms of planning and delivery and were therefore put off from delivering programmes:

Because of the nature of HENRY and the amount of planning and setting up, and reading, and the length of it, it does impact on us as staffing because in children’s centres, and you may well know we have very limited staffing at any of our centres (HENRY alone manager)

Moreover, the priority placed on HENRY itself was mixed which may have influenced the priority placed on engaging parents to the programme, as some managers described how HENRY was just one programme on offer among a variety of other services and initiatives:

Across the cluster we were following sort of the new initiative of the ‘eat better start better’ guidelines, that was introduced a few years ago, so that then became our focus more rather than HENRY (HENRY + optimisation intervention manager)

## Discussion

This trial, delivered at scale across 20 local government areas of the UK, found no evidence that an implementation optimisation intervention improved parental enrolment and attendance at an obesity prevention programme. Previous studies have mostly evaluated interventions directly aimed at parents, such as financial incentives [39, 53, 54] and promotional strategies [55, 56], with only limited effects [55–57]. To our knowledge, this is the only implementation optimisation intervention aimed at changing behaviours across multiple organisational levels to promote parental enrolment and attendance, recognising the necessity of infrastructure, resources and skills to optimise enrolment and attendance.

Our process evaluation highlighted how contextual factors undermined the ability of sites to prioritise engagement with the implementation optimisation intervention. These factors included reduced funding and the associated reductions in service capacity, amalgamation of services, and threats to jobs. A similar trial exploring the implementation of a fire injury prevention intervention in children’s centres in 2012 reported similar results [58], with a nested qualitative study also describing that uncertainties surrounding the future of children’s centres and imminent restructuring impeded its implementation [59].

Given the complexity of this setting, research undertaken in early years settings is inevitability challenging. It remains plausible that bespoke or locally adapted interventions that are responsive to local context and collaboratively developed with stakeholders may achieve greater implementation fidelity [21, 60] [52]. However, given major cuts to children’s centre services during the period of this study, where overall funding fell by 64% from 2010 to 2018 [61], it seems unlikely that any intervention would have had demonstrable effects.

The process evaluation sheds light on a potential lack of engagement with the existing HENRY programme due to limited capacity and resources to deliver the programme. This resulted in a reduced priority on promoting enrolment and attendance to the programme. The priority placed on the delivery of HENRY programmes or similar may be influenced by stakeholder perceptions of whether the programme can demonstrate an effect [62], but these data are rarely available for public health obesity prevention programmes delivered at scale. In this study, we applied a novel approach to evidence generation through the conduct of a comprehensive early-phase (evidentiary) intervention enhancement and evaluation [13] prior to testing the feasibility of assessing the effectiveness of HENRY [15]. This is in contrast to conventional implementation research, which would usually be conducted following definitive randomised evaluations to determine clinical effectiveness (in this case, childhood obesity prevention). This novel approach ensures that factors which limit trial outcomes, such as low adherence, are minimised prior to dedicating the resources required to conduct a large trial which may identify no evidence of effectiveness, perhaps as a result of poor compliance. As the HENRY implementation optimisation intervention was informed by an ethnography study (where existing HENRY engagement practices were observed), the wider literature on promoting enrolment and attendance, and the experience and expertise of the intervention development team (including stakeholders, such as a HENRY representative a HENRY facilitator), it was anticipated that some target behaviours would have been performed by control centres in line with usual practice or that other strategies would have been used outside of target behaviours to promote enrolment and attendance to HENRY. This was mitigated in the study design by using randomisation and baseline level of engagement as a stratification variable. Participation bias was also considered and minimised by the use of an opt-out approach to local authority recruitment along with the inclusion of a diverse range of providers with varying baseline engagement levels. As the implementation optimisation intervention sought to persuade stakeholders to perform a specific set of behaviours to promote enrolment and attendance to HENRY, the number of centres performing target behaviours would be expected to be significantly greater in the intervention arm. Thus, similarities between the arms suggest that the intervention did not instigate behaviour change as proposed. However, given that the optimisation intervention could not be delivered as planned given such an unfavourable context, it is difficult to draw definitive conclusions on its effectiveness.

There remains a strong need for approaches to improve engagement with public health programmes delivered in community settings to optimise their impact on family and child outcomes [63, 64]. Programmes delivered in children’s centres offer the potential to reach families living in the most deprived areas, highlighting their potential as a public health delivery setting. However, programmes like HENRY and strategies to optimise their implementation are unlikely to be viable in the absence of sufficient resources, management commitment and organisational stability [65]. Ideally, practical implementation considerations need to be integrated during the design phase of public health programmes and interventions to optimise their implementation to (ideally) make them more robust and sustainable in unfavourable fiscal and organisational climates. Having a good understanding of the setting is also critical. We developed our approach based on an ethnography, including extensive interviews and discussion with stakeholders (identifying barriers and opportunities improved implementation of interventions [21] followed by the use of a co-design approach for theory-based intervention development [22]). However, we would recommend that future research considers how to factor in how organisations can enhance levels of resilience to deal with unfavourable contexts, such as major restructuring of the way organisations work or funding cuts.

This trial tested a novel implementation optimisation intervention which was developed using a theoretical framework, primary research in children’s centres, and the wider literature on engagement methods. The use of routine data to measure outcomes allowed for a greater breadth of recruitment and minimised the commitment required by intervention teams, particularly during a time when capacity within local authorities and children’s centres was already stretched. Process evaluation shed light on the factors that likely hindered the impact of the intervention, particularly the impact of the wider environmental changes in the setting which resulted in a reduction in engagement in both the intervention and control arms of the trial. Not only does this contribute to the literature on engagement, it also provides valuable lessons for undertaking research within early years settings.

Although the failure of the trial to detect any impact of the implementation intervention has been attributed to poor intervention fidelity and contamination, we are unable to confirm or deny its potential effectiveness even under an assumption that fidelity was high. Despite the robust intervention development and trial design, political and austerity measures disrupted planned implementation beyond our control. Given that our primary outcome data were parent engagement, missing data from children’s centres were considered to indicate a lack of engagement; thus, imputation was not appropriate. While we met our recruitment target, this lack of data inevitably reduced our statistical power and resulted in wide variability. It is also possible that the disruptions influenced the ability and priority of centres to collect and share data. It is possible that more parents engaged in the HENRY programme than were recorded. Furthermore, HENRY engagement initiatives occurring in control areas may have ‘diluted’ the intervention effects by which parents attending centres in the control condition received some of the recommended strategies which may have influenced enrolment or completion. Our process evaluation highlighted the difficulties in maintaining a control condition in a pragmatic trial where centres in both trial arms sought to enhance engagement to HENRY to maximise value for money. Inviting centres that were HENRY naïve to participate in the trial may have minimised the sharing of knowledge and ideas on ways to promote engagement prior to the study.

## Supplementary Information

**Additional file 1.** Additional tables

## Data Availability

The data that support the findings of this study are available from HENRY central office but restrictions apply to the availability of these data, which were used under license for the current study, and so are not publicly available. Data are however available from the authors upon reasonable request and with permission of the HENRY central office.

## Abbreviations

cRCT: Cluster randomised controlled trial
CTRU: Clinical Trials Research Unit
OFTEN: Optimising Family EngagemenT in HENRY
*ICC*: Intra-cluster correlation coefficient
