# Supplementary material for: A cluster RCT and process evaluation of an implementation optimisation intervention to promote parental engagement enrolment and attendance in a childhood obesity prevention programme: results of the Optimising Family Engagement in HENRY (OFTEN) trial
Source: Trials. 2021 Nov 5;22:773. doi: 10.1186/s13063-021-05757-w (PMC8569980; doi:10.1186/s13063-021-05757-w)
Supplement: Supplementary file 1 — Additional file 1. Additional tables [file 13063_2021_5757_MOESM1_ESM.docx]

**Additional tables**

Additional Table 1: Power calculations for various intervention effect estimates and a fixed sample size (144 children’s centres, 24 local authorities^a^)

| **Outcome in the control** | **Percentage point increase in intervention** | **Power for ICC=0.1, α=0.05** | **Power for ICC=0.05, α=0.05** | **Power for ICC=0.1, α=0.025** | **Power for ICC=0.05, α=0.025** |
| --- | --- | --- | --- | --- | --- |
| Recruitment (≥8 parents per programme) | | | |  |  |
| 55% | 5% | 6% | 7% | 3% | 4% |
|  | 10% | 15% | 18% | 9% | 11% |
|  | 15% | 29% | 35% | 20% | 25% |
|  | 20% | 49% | 58% | 38% | 46% |
|  | 25% | 70% | 79% | 59% | 70% |
|  | 30% | 87% | 93% | 79% | 88% |
| Retention (≥75% of parents attending 5/8 sessions) | | | |  |  |
| 50% | 5% | 6% | 7% | 3% | 4% |
|  | 10% | 15% | 17% | 9% | 11% |
|  | 15% | 28% | 34% | 19% | 24% |
|  | 20% | 47% | 55% | 36% | 44% |
|  | 25% | 67% | 76% | 56% | 66% |
|  | 30% | 84% | 91% | 76% | 85% |

^a^Assumes a coefficient of variation of 0.54

Additional Table 2. Participant gender at pre-randomisation by Local Authority

|  | Male N (%) | Female N (%) | Prefer not to say N (%) | Missing N (%) | Total N (%) |
| --- | --- | --- | --- | --- | --- |
| LA |  |  |  |  |  |
| LA1 | 2 (9.1%) | 22 (5.1%) | 0 (0.0%) | 19 (4.6%) | 43 (4.9%) |
| LA2 | 0 (0.0%) | 12 (2.8%) | 0 (0.0%) | 12 (2.9%) | 24 (2.7%) |
| LA3 | 0 (0.0%) | 5 (1.2%) | 0 (0.0%) | 4 (1.0%) | 9 (1.0%) |
| LA4 | 2 (9.1%) | 4 (0.9%) | 1 (12.5%) | 26 (6.2%) | 33 (3.7%) |
| LA5 | 3 (13.6%) | 31 (7.1%) | 0 (0.0%) | 35 (8.4%) | 69 (7.8%) |
| LA6 | 1 (4.5%) | 12 (2.8%) | 0 (0.0%) | 38 (9.1%) | 51 (5.8%) |
| LA7 | 0 (0.0%) | 7 (1.6%) | 0 (0.0%) | 9 (2.2%) | 16 (1.8%) |
| LA8 | 0 (0.0%) | 50 (11.5%) | 1 (12.5%) | 66 (15.8%) | 117 (13.3%) |
| LA9 | 0 (0.0%) | 20 (4.6%) | 0 (0.0%) | 12 (2.9%) | 32 (3.6%) |
| LA10 | 0 (0.0%) | 38 (8.8%) | 1 (12.5%) | 26 (6.2%) | 65 (7.4%) |
| LA11 | 0 (0.0%) | 3 (0.7%) | 0 (0.0%) | 6 (1.4%) | 9 (1.0%) |
| LA12 | 1 (4.5%) | 22 (5.1%) | 0 (0.0%) | 2 (0.5%) | 25 (2.8%) |
| LA13 | 4 (18.2%) | 15 (3.5%) | 2 (25.0%) | 10 (2.4%) | 31 (3.5%) |
| LA14 | 2 (9.1%) | 35 (8.1%) | 0 (0.0%) | 31 (7.4%) | 68 (7.7%) |
| LA15 | 1 (4.5%) | 21 (4.8%) | 0 (0.0%) | 6 (1.4%) | 28 (3.2%) |
| LA16 | 0 (0.0%) | 15 (3.5%) | 0 (0.0%) | 41 (9.8%) | 56 (6.4%) |
| LA17 | 1 (4.5%) | 7 (1.6%) | 0 (0.0%) | 12 (2.9%) | 20 (2.3%) |
| LA18 | 2 (9.1%) | 49 (11.3%) | 2 (25.0%) | 17 (4.1%) | 70 (7.9%) |
| LA19 | 3 (13.6%) | 43 (9.9%) | 0 (0.0%) | 41 (9.8%) | 87 (9.9%) |
| LA20 | 0 (0.0%) | 23 (5.3%) | 1 (12.5%) | 4 (1.0%) | 28 (3.2%) |
| Total | 22 (100%) | 434 (100%) | 8 (100%) | 417 (100%) | 881 (100%) |

Additional Table 3. Participant age at pre-randomisation by Local Authority

|  | Under 18 N (%) | 18-25 N (%) | 25-64 N (%) | 65+ N (%) | Prefer not to say N (%) | Missing N (%) | Total N (%) |
| --- | --- | --- | --- | --- | --- | --- | --- |
| LA |  |  |  |  |  |  |  |
| LA1 | 0 (0.0%) | 11 (13.4%) | 11 (3.0%) | 2 (50.0%) | 0 (0.0%) | 19 (4.6%) | 43 (4.9%) |
| LA2 | 0 (0.0%) | 0 (0.0%) | 12 (3.2%) | 0 (0.0%) | 0 (0.0%) | 12 (2.9%) | 24 (2.7%) |
| LA3 | 0 (0.0%) | 3 (3.7%) | 2 (0.5%) | 0 (0.0%) | 0 (0.0%) | 4 (1.0%) | 9 (1.0%) |
| LA4 | 0 (0.0%) | 1 (1.2%) | 5 (1.4%) | 0 (0.0%) | 1 (12.5%) | 26 (6.2%) | 33 (3.7%) |
| LA5 | 0 (0.0%) | 7 (8.5%) | 26 (7.0%) | 0 (0.0%) | 1 (12.5%) | 35 (8.4%) | 69 (7.8%) |
| LA6 | 0 (0.0%) | 2 (2.4%) | 11 (3.0%) | 0 (0.0%) | 0 (0.0%) | 38 (9.1%) | 51 (5.8%) |
| LA7 | 0 (0.0%) | 0 (0.0%) | 7 (1.9%) | 0 (0.0%) | 0 (0.0%) | 9 (2.2%) | 16 (1.8%) |
| LA8 | 0 (0.0%) | 4 (4.9%) | 47 (12.7%) | 0 (0.0%) | 0 (0.0%) | 66 (15.8%) | 117 (13.3%) |
| LA9 | 0 (0.0%) | 5 (6.1%) | 15 (4.1%) | 0 (0.0%) | 0 (0.0%) | 12 (2.9%) | 32 (3.6%) |
| LA10 | 0 (0.0%) | 6 (7.3%) | 31 (8.4%) | 0 (0.0%) | 2 (25.0%) | 26 (6.2%) | 65 (7.4%) |
| LA11 | 0 (0.0%) | 0 (0.0%) | 3 (0.8%) | 0 (0.0%) | 0 (0.0%) | 6 (1.4%) | 9 (1.0%) |
| LA12 | 0 (0.0%) | 1 (1.2%) | 20 (5.4%) | 1 (25.0%) | 1 (12.5%) | 2 (0.5%) | 25 (2.8%) |
| LA13 | 0 (0.0%) | 5 (6.1%) | 15 (4.1%) | 0 (0.0%) | 1 (12.5%) | 10 (2.4%) | 31 (3.5%) |
| LA14 | 0 (0.0%) | 6 (7.3%) | 30 (8.1%) | 1 (25.0%) | 0 (0.0%) | 31 (7.4%) | 68 (7.7%) |
| LA15 | 0 (0.0%) | 5 (6.1%) | 17 (4.6%) | 0 (0.0%) | 0 (0.0%) | 6 (1.4%) | 28 (3.2%) |
| LA16 | 0 (0.0%) | 2 (2.4%) | 13 (3.5%) | 0 (0.0%) | 0 (0.0%) | 41 (9.8%) | 56 (6.4%) |
| LA17 | 0 (0.0%) | 0 (0.0%) | 8 (2.2%) | 0 (0.0%) | 0 (0.0%) | 12 (2.9%) | 20 (2.3%) |
| LA18 | 0 (0.0%) | 15 (18.3%) | 37 (10.0%) | 0 (0.0%) | 1 (12.5%) | 17 (4.1%) | 70 (7.9%) |
| LA19 | 0 (0.0%) | 7 (8.5%) | 39 (10.5%) | 0 (0.0%) | 0 (0.0%) | 41 (9.8%) | 87 (9.9%) |
| LA20 | 0 (0.0%) | 2 (2.4%) | 21 (5.7%) | 0 (0.0%) | 1 (12.5%) | 4 (1.0%) | 28 (3.2%) |
| Total | 0 (0.0%) | 82 (100%) | 370 (100%) | 4 (100%) | 8 (100%) | 417 (100%) | 881 (100%) |

Additional Table 4. Number and ages of children at pre-randomisation by Local Authority

|  | Number of children^a^ | Number of children aged < 1yrs^b^ | Number of children aged 1yrs^b^ | Number of children aged 2yrs^b^ | Number of children aged 3yrs^b^ | Number of children aged 4yrs^b^ | Number of children aged 5yrs^b^ |
| --- | --- | --- | --- | --- | --- | --- | --- |
| LA |  |  |  |  |  |  |  |
| LA1 | 26 (5.1%) | 9 (6.8%) | 6 (4.6%) | 3 (3.9%) | 2 (2.9%) | 5 (7.7%) | 1 (2.6%) |
| LA2 | 13 (2.5%) | 5 (3.8%) | 4 (3.1%) | 1 (1.3%) | 1 (1.4%) | 2 (3.1%) | 0 (0.0%) |
| LA3 | 7 (1.4%) | 2 (1.5%) | 1 (0.8%) | 1 (1.3%) | 2 (2.9%) | 1 (1.5%) | 0 (0.0%) |
| LA4 | 12 (2.3%) | 3 (2.3%) | 4 (3.1%) | 2 (2,6%) | 2 (2.9%) | 0 (0.0%) | 1 (2.6%) |
| LA5 | 28 (5.4%) | 9 (6.8%) | 8 (6.2%) | 1 (1.3%) | 4 (5.7%) | 3 (4.6%) | 3 (7.7%) |
| LA6 | 9 (1.8%) | 4 (3.0%) | 4 (3.1%) | 1 (1.3%) | 0 (0.0%) | 0 (0.0%) | 0 (0.0%) |
| LA7 | 10 (1.9%) | 5 (3.8%) | 0 (0.0%) | 3 (3.9%) | 0 (0.0%) | 1 (1.5%) | 1 (2.6%) |
| LA8 | 61 (11.9%) | 16 (12.0%) | 10 (7.7%) | 14 (18.2%) | 8 (11.4%) | 8 (12.3%) | 5 (12.8%) |
| LA9 | 25 (4.9%) | 12 (9.0%) | 0 (0.0%) | 4 (5.2%) | 5 (7.1%) | 2 (3.1%) | 2 (5.1%) |
| LA10 | 59 (11.5%) | 7 (5.3%) | 15 (11.5%) | 7 (9.1%) | 11 (15.7%) | 12 (18.5%) | 7 (17.9%) |
| LA11 | 3 (0.6%) | 0 (0.0%) | 1 (0.8%) | 0 (0.0%) | 2 (2.9%) | 0 (0.0%) | 0 (0.0%) |
| LA12 | 27 (5.3%) | 6 (4.5%) | 5 (3.8%) | 5 (6.5%) | 5 (7.1%) | 5 (7.7%) | 1 (2.6%) |
| LA13 | 17 (3.3%) | 9 (6.8%) | 3 (2.3%) | 1 (1.3%) | 0 (0.0%) | 2 (3.1%) | 2 (5.1%) |
| LA14 | 31 (6.0%) | 9 (6.8%) | 6 (4.6%) | 6 (7.8%) | 6 (8.6%) | 4 (6.2%) | 0 (0.0%) |
| LA15 | 32 (6.2%) | 5 (3.8%) | 12 (9.2%) | 7 (9.1%) | 3 (4.3%) | 1 (1.5%) | 4 (10.3%) |
| LA16 | 14 (2.7%) | 1 (0.8%) | 5 (3.8%) | 2 (2.6%) | 3 (4.3%) | 2 (3.1%) | 1 (2.6%) |
| LA17 | 8 (1.6%) | 3 (2.3%) | 4 (3.1%) | 1 (1.3%) | 0 (0.0%) | 0 (0.0%) | 0 (0.0%) |
| LA18 | 45 (8.8%) | 10 (7.5%) | 24 (18.5%) | 4 (5.2%) | 3 (4.3%) | 3 (4.6%) | 1 (2.6%) |
| LA19 | 54 (10.5%) | 12 (9.0%) | 107.7% | 10 (13.0%) | 8 (11.4%) | 8 (12.3%) | 6 (15.4%) |
| LA20 | 33 (6.4%) | 6 (4.5%) | 8 (6.2%) | 4 (5.2%) | 5 (7.1%) | 6 (9.2%) | 4 (10.3%) |
| Total | 514 (100%) | 133 (100%) | 130 (100%) | 77 (100%) | 70 (100%) | 65 (100%) | 39 (100%) |

^a^Collected as the number of children of each age per parent, numbers reported therefore total more than the number of parents at baseline.

^b^Data was missing for 417 parents.

Additional Table 5. Participant ethnicity at pre-registration by Local Authority

|  | African N (%) | Arab N (%) | Bangadeshi N (%) | Black UK N (%) | Caribbean N (%) | Chinese N (%) | English / Scottish / Welsh / Northern Irish / UK N (%) | Gypsy or Irish Traveller N (%) | Indian N (%) |
| --- | --- | --- | --- | --- | --- | --- | --- | --- | --- |
| LA |  |  |  |  |  |  |  |  |  |
| LA1 | 0 (0.0%) | 0 (0.0%) | 0 (0.0%) | 0 (0.0%) | 0 (0.0%) | 0 (0.0%) | 23 (7.0%) | 0 (0.0%) | 0 (0.0%) |
| LA2 | 0 (0.0%) | 0 (0.0%) | 0 (0.0%) | 0 (0.0%) | 0 (0.0%) | 1 (33.3%) | 7 (2.1%) | 0 (0.0%) | 0 (0.0%) |
| LA3 | 0 (0.0%) | 0 (0.0%) | 0 (0.0%) | 1 (16.7%) | 0 (0.0%) | 0 (0.0%) | 4 (1.2%) | 0 (0.0%) | 0 (0.0%) |
| LA4 | 0 (0.0%) | 0 (0.0%) | 0 (0.0%) | 0 (0.0%) | 0 (0.0%) | 0 (0.0%) | 5 (1.5%) | 0 (0.0%) | 0 (0.0%) |
| LA5 | 1 (4.5%) | 0 (0.0%) | 1 (9.1%) | 0 (0.0%) | 0 (0.0%) | 0 (0.0%) | 22 (6.7%) | 0 (0.0%) | 0 (0.0%) |
| LA6 | 1 (4.5%) | 0 (0.0%) | 0 (0.0%) | 1 (16.7%) | 0 (0.0%) | 0 (0.0%) | 6 (1.8%) | 0 (0.0%) | 0 (0.0%) |
| LA7 | 0 (0.0%) | 0 (0.0%) | 0 (0.0%) | 0 (0.0%) | 0 (0.0%) | 0 (0.0%) | 0 (0.0%) | 0 (0.0%) | 0 (0.0%) |
| LA8 | 13 (59.1%) | 0 (0.0%) | 8 (72.7%) | 1 (16.7%) | 6 (85.7%) | 0 (0.0%) | 8 (2.4%) | 0 (0.0%) | 3 (30.0%) |
| LA9 | 0 (0.0%) | 0 (0.0%) | 0 (0.0%) | 0 (0.0%) | 0 (0.0%) | 0 (0.0%) | 19 (5.8%) | 0 (0.0%) | 0 (0.0%) |
| LA10 | 0 (0.0%) | 0 (0.0%) | 0 (0.0%) | 0 (0.0%) | 0 (0.0%) | 1 (33.3%) | 36 (11.0%) | 0 (0.0%) | 0 (0.0%) |
| LA11 | 0 (0.0%) | 0 (0.0%) | 0 (0.0%) | 0 (0.0%) | 0 (0.0%) | 0 (0.0%) | 2 (0.6%) | 0 (0.0%) | 0 (0.0%) |
| LA12 | 5 (22.7%) | 0 (0.0%) | 0 (0.0%) | 1 (16.7%) | 1 (14.3%) | 1 (33.3%) | 4 (1.2%) | 0 (0.0%) | 1 (10.0%) |
| LA13 | 0 (0.0%) | 0 (0.0%) | 0 (0.0%) | 0 (0.0%) | 0 (0.0%) | 0 (0.0%) | 20 (6.1%) | 0 (0.0%) | 0 (0.0%) |
| LA14 | 0 (0.0%) | 0 (0.0%) | 0 (0.0%) | 2 (33.3%) | 0 (0.0%) | 0 (0.0%) | 31 (9.5%) | 0 (0.0%) | 1 (10.0%) |
| LA15 | 1 (4.5%) | 0 (0.0%) | 0 (0.0%) | 0 (0.0%) | 0 (0.0%) | 0 (0.0%) | 17 (5.2%) | 1 (100.0%) | 1 (10.0%) |
| LA16 | 0 (0.0%) | 0 (0.0%) | 0 (0.0%) | 0 (0.0%) | 0 (0.0%) | 0 (0.0%) | 14 (4.3%) | 0 (0.0%) | 0 (0.0%) |
| LA17 | 0 (0.0%) | 0 (0.0%) | 0 (0.0%) | 0 (0.0%) | 0 (0.0%) | 0 (0.0%) | 6 (1.8%) | 0 (0.0%) | 0 (0.0%) |
| LA18 | 0 (0.0%) | 1 (100.0%) | 0 (0.0%) | 0 (0.0%) | 0 (0.0%) | 0 (0.0%) | 44 (13.5%) | 0 (0.0%) | 0 (0.0%) |
| LA19 | 1 (4.5%) | 0 (0.0%) | 2 (18.2%) | 0 (0.0%) | 0 (0.0%) | 0 (0.0%) | 37 (11.3%) | 0 (0.0%) | 4 (40.0%) |
| LA20 | 0 (0.0%) | 0 (0.0%) | 0 (0.0%) | 0 (0.0%) | 0 (0.0%) | 0 (0.0%) | 22 (6.7%) | 0 (0.0%) | 0 (0.0%) |
| Total | 22 (100%) | 1 (100%) | 11 (100%) | 6 (100%) | 7 (100%) | 3 (100%) | 327 (100%) | 1 (100%) | 10 (100%) |

Additional Table 5. Participant ethnicity at pre-registration by Local Authority (ctd.)

|  | Irish N (%) | Mixed ethnic background N (%) | Pakistani N (%) | Any other Asian background N (%) | Any other Black / African / Caribbean background N (%) | Any other White background N (%) | Any other ethnic group N (%) | Prefer not to say N (%) | Missing N (%) | Total N (%) |
| --- | --- | --- | --- | --- | --- | --- | --- | --- | --- | --- |
| LA |  |  |  |  |  |  |  |  |  |  |
| LA1 | 0 (0.0%) | 0 (0.0%) | 0 (0.0%) | 0 (0.0%) | 0 (0.0%) | 0 (0.0%) | 0 (0.0%) | 1 (5.9%) | 19 (4.6%) | 43 (4.9%) |
| LA2 | 0 (0.0%) | 0 (0.0%) | 1 (6.7%) | 0 (0.0%) | 0 (0.0%) | 3 (11.5%) | 0 (0.0%) | 0 (0.0%) | 12 (2.9%) | 24 (2.7%) |
| LA3 | 0 (0.0%) | 0 (0.0%) | 0 (0.0%) | 0 (0.0%) | 0 (0.0%) | 0 (0.0%) | 0 (0.0%) | 0 (0.0%) | 4 (1.0%) | 9 (1.0%) |
| LA4 | 0 (0.0%) | 0 (0.0%) | 1 (6.7%) | 0 (0.0%) | 0 (0.0%) | 0 (0.0%) | 0 (0.0%) | 1 (5.9%) | 26 (6.2%) | 33 (3.7%) |
| LA5 | 0 (0.0%) | 0 (0.0%) | 9 (60.0%) | 0 (0.0%) | 0 (0.0%) | 0 (0.0%) | 0 (0.0%) | 1 (5.9%) | 35 (8.4%) | 69 (7.8%) |
| LA6 | 0 (0.0%) | 0 (0.0%) | 0 (0.0%) | 0 (0.0%) | 1 (50.0%) | 2 (7.7%) | 2 (33.3%) | 0 (0.0%) | 38 (9.1%) | 51 (5.8%) |
| LA7 | 0 (0.0%) | 0 (0.0%) | 0 (0.0%) | 0 (0.0%) | 0 (0.0%) | 0 (0.0%) | 0 (0.0%) | 7 (41.2%) | 9 (2.2%) | 16 (1.8%) |
| LA8 | 0 (0.0%) | 1 (25.0%) | 0 (0.0%) | 1 (16.7%) | 1 (50.0%) | 6 (23.1%) | 3 (50.0%) | 0 (0.0%) | 66 (15.8%) | 117 (13.3%) |
| LA9 | 0 (0.0%) | 0 (0.0%) | 0 (0.0%) | 0 (0.0%) | 0 (0.0%) | 1 (3.8%) | 0 (0.0%) | 0 (0.0%) | 12 (2.9%) | 32 (3.6%) |
| LA10 | 0 (0.0%) | 1 (25.0%) | 0 (0.0%) | 0 (0.0%) | 0 (0.0%) | 1 (3.8%) | 0 (0.0%) | 0 (0.0%) | 26 (6.2%) | 65 (7.4%) |
| LA11 | 0 (0.0%) | 0 (0.0%) | 0 (0.0%) | 0 (0.0%) | 0 (0.0%) | 1 (3.8%) | 0 (0.0%) | 0 (0.0%) | 6 (1.4%) | 9 (1.0%) |
| LA12 | 0 (0.0%) | 1 (25.0%) | 0 (0.0%) | 3 (50.0%) | 0 (0.0%) | 4 (15.4%) | 0 (0.0%) | 2 (11.8%) | 2 (0.5%) | 25 (2.8%) |
| LA13 | 0 (0.0%) | 0 (0.0%) | 0 (0.0%) | 0 (0.0%) | 0 (0.0%) | 0 (0.0%) | 0 (0.0%) | 1 (5.9%) | 10 (2.4%) | 31 (3.5%) |
| LA14 | 0 (0.0%) | 0 (0.0%) | 2 (13.3%) | 1 (16.7%) | 0 (0.0%) | 0 (0.0%) | 0 (0.0%) | 0 (0.0%) | 31 (7.4%) | 68 (7.7%) |
| LA15 | 0 (0.0%) | 0 (0.0%) | 1 (6.7%) | 0 (0.0%) | 0 (0.0%) | 0 (0.0%) | 0 (0.0%) | 1 (5.9%) | 6 (1.4%) | 28 (3.2%) |
| LA16 | 0 (0.0%) | 0 (0.0%) | 0 (0.0%) | 0 (0.0%) | 0 (0.0%) | 1 (3.8%) | 0 (0.0%) | 0 (0.0%) | 41 (9.8%) | 56 (6.4%) |
| LA17 | 0 (0.0%) | 0 (0.0%) | 0 (0.0%) | 0 (0.0%) | 0 (0.0%) | 2 (7.7%) | 0 (0.0%) | 0 (0.0%) | 12 (2.9%) | 20 (2.3%) |
| LA18 | 0 (0.0%) | 0 (0.0%) | 1 (6.7%) | 1 (16.7%) | 0 (0.0%) | 4 (15.4%) | 1 (16.7%) | 1 (5.9%) | 17 (4.1%) | 70 (7.9%) |
| LA19 | 0 (0.0%) | 1 (25.0%) | 0 (0.0%) | 0 (0.0%) | 0 (0.0%) | 0 (0.0%) | 0 (0.0%) | 1 (5.9%) | 41 (9.8%) | 87 (9.9%) |
| LA20 | 0 (0.0%) | 0 (0.0%) | 0 (0.0%) | 0 (0.0%) | 0 (0.0%) | 1 (3.8%) | 0 (0.0%) | 1 (5.9%) | 4 (1.0%) | 28 (3.2%) |
| Total | 0 (0.0%) | 4 (100%) | 15 (100%) | 6 (100%) | 2 (100%) | 26 (100%) | 6 (100%) | 17 (100%) | 417 (100%) | 881 (100%) |

Additional Table 6. How did the participant hear about the HENRY programme at pre-randomisation by Local Authority

|  | Family and friends N (%) | Leaflet N (%) | Poster N (%) | Professional N (%) | Website N (%) | Other N (%) | Missing N (%) | Total N (%) |
| --- | --- | --- | --- | --- | --- | --- | --- | --- |
| LA |  |  |  |  |  |  |  |  |
| LA1 | 0 (0.0%) | 0 (0.0%) | 0 (0.0%) | 0 (0.0%) | 0 (0.0%) | 0 (0.0%) | 43 (6.2%) | 43 (4.9%) |
| LA2 | 0 (0.0%) | 0 (0.0%) | 0 (0.0%) | 2 (1.8%) | 0 (0.0%) | 5 (17.9%) | 17 (2.4%) | 24 (2.7%) |
| LA3 | 0 (0.0%) | 0 (0.0%) | 0 (0.0%) | 4 (3.7%) | 0 (0.0%) | 1 (3.6%) | 4 (0.6%) | 9 (1.0%) |
| LA4 | 0 (0.0%) | 0 (0.0%) | 0 (0.0%) | 3 (2.8%) | 0 (0.0%) | 0 (0.0%) | 30 (4.3%) | 33 (3.7%) |
| LA5 | 1 (7.1%) | 3 (12.0%) | 0 (0.0%) | 4 (3.7%) | 0 (0.0%) | 4 (14.3%) | 57 (8.2%) | 69 (7.8%) |
| LA6 | 0 (0.0%) | 0 (0.0%) | 0 (0.0%) | 0 (0.0%) | 0 (0.0%) | 0 (0.0%) | 51 (7.3%) | 51 (5.8%) |
| LA7 | 0 (0.0%) | 0 (0.0%) | 0 (0.0%) | 0 (0.0%) | 0 (0.0%) | 0 (0.0%) | 16 (2.3%) | 16 (1.8%) |
| LA8 | 2 (14.3%) | 6 (24.0%) | 1 (14.3%) | 14 (12.8%) | 0 (0.0%) | 6 (21.4%) | 88 (12.7%) | 117 (13.3%) |
| LA9 | 0 (0.0%) | 4 (16.0%) | 1 (14.3%) | 11 (10.1%) | 1 (33.3%) | 1 (3.6%) | 14 (2.0%) | 32 (3.6%) |
| LA10 | 1 (7.1%) | 6 (24.0%) | 1 (14.3%) | 15 (13.8%) | 1 (33.3%) | 2 (7.1%) | 39 (5.6%) | 65 (7.4%) |
| LA11 | 0 (0.0%) | 0 (0.0%) | 0 (0.0%) | 0 (0.0%) | 0 (0.0%) | 0 (0.0%) | 9 (1.3%) | 9 (1.0%) |
| LA12 | 1 (7.1%) | 1 (4.0%) | 2 (28.6%) | 12 (11.0%) | 0 (0.0%) | 7 (25.0%) | 2 (0.3%) | 25 (2.8%) |
| LA13 | 3 (21.4%) | 2 (8.0%) | 0 (0.0%) | 14 (12.8%) | 1 (33.3%) | 1 (3.6%) | 10 (1.4%) | 31 (3.5%) |
| LA14 | 0 (0.0%) | 0 (0.0%) | 0 (0.0%) | 0 (0.0%) | 0 (0.0%) | 0 (0.0%) | 68 (9.8%) | 68 (7.7%) |
| LA15 | 0 (0.0%) | 0 (0.0%) | 0 (0.0%) | 7 (6.4%) | 0 (0.0%) | 0 (0.0%) | 21 (3.0%) | 28 (3.2%) |
| LA16 | 2 (14.3%) | 0 (0.0%) | 0 (0.0%) | 6 (5.5%) | 0 (0.0%) | 0 (0.0%) | 48 (6.9%) | 56 (6.4%) |
| LA17 | 0 (0.0%) | 0 (0.0%) | 0 (0.0%) | 1 (0.9%) | 0 (0.0%) | 1 (3.6%) | 18 (2.6%) | 20 (2.3%) |
| LA18 | 2 (14.3%) | 2 (8.0%) | 0 (0.0%) | 8 (7.3%) | 0 (0.0%) | 0 (0.0%) | 58 (8.3%) | 70 (7.9%) |
| LA19 | 2 (14.3%) | 1 (4.0%) | 2 (28.6%) | 8 (7.3%) | 0 (0.0%) | 0 (0.0%) | 74 (10.6%) | 87 (9.9%) |
| LA20 | 0 (0.0%) | 0 (0.0%) | 0 (0.0%) | 0 (0.0%) | 0 (0.0%) | 0 (0.0%) | 28 (4.0%) | 28 (3.2%) |
| Total | 14 (100%) | 25 (100%) | 7 (100%) | 109 (100%) | 3 (100%) | 28 (100%) | 695 (100%) | 881 (100%) |

Additional Table 7. Participant gender at follow-up by Local Authority

|  | Male N (%) | Female N (%) | Prefer not to say N (%) | Missing N (%) | Total N (%) |
| --- | --- | --- | --- | --- | --- |
| LA |  |  |  |  |  |
| LA1 | 0 (0.0%) | 10 (4.3%) | 0 (0.0%) | 4 (2.1%) | 14 (3.2%) |
| LA2 | 0 (0.0%) | 5 (2.2%) | 0 (0.0%) | 0 (0.0%) | 5 (1.2%) |
| LA3^a^ | 0 (0.0%) | 0 (0.0%) | 0 (0.0%) | 0 (0.0%) | 0 (0.0%) |
| LA4 | 2 (18.2%) | 3 (1.3%) | 0 (0.0%) | 20 (10.6%) | 25 (5.8%) |
| LA5 | 0 (0.0%) | 30 (12.9%) | 0 (0.0%) | 28 (14.9%) | 58 (13.4%) |
| LA6 | 1 (9.1%) | 19 (8.2%) | 0 (0.0%) | 14 (7.4%) | 34 (7.9%) |
| LA7 | 0 (0.0%) | 0 (0.0%) | 0 (0.0%) | 6 (3.2%) | 6 (1.4%) |
| LA8 | 1 (9.1%) | 44 (19.0%) | 2 (100.0%) | 34 (18.1%) | 81 (18.7%) |
| LA9 | 2 (18.2%) | 13 (5.6%) | 0 (0.0%) | 11 (5.9%) | 26 (6.0%) |
| LA10 | 0 (0.0%) | 7 (3.0%) | 0 (0.0%) | 2 (1.1%) | 9 (2.1%) |
| LA11^a^ | 0 (0.0%) | 0 (0.0%) | 0 (0.0%) | 0 (0.0%) | 0 (0.0%) |
| LA12 | 1 (9.1%) | 17 (7.3%) | 0 (0.0%) | 4 (2.1%) | 22 (5.1%) |
| LA13 | 2 (18.2%) | 13 (5.6%) | 0 (0.0%) | 4 (2.1%) | 19 (4.4%) |
| LA14 | 0 (0.0%) | 7 (3.0%) | 0 (0.0%) | 4 (2.1%) | 11 (2.5%) |
| LA15 | 0 (0.0%) | 3 (1.3%) | 0 (0.0%) | 5 (2.7%) | 8 (1.8%) |
| LA16 | 2 (18.2%) | 19 (8.2%) | 0 (0.0%) | 19 (10.1%) | 40 (9.2%) |
| LA17 | 0 (0.0%) | 10 (4.3%) | 0 (0.0%) | 4 (2.1%) | 14 (3.2%) |
| LA18 | 0 (0.0%) | 23 (9.9%) | 0 (0.0%) | 20 (10.6%) | 43 (9.9%) |
| LA19 | 0 (0.0%) | 9 (3.9%) | 0 (0.0%) | 9 (4.8%) | 18 (4.2%) |
| LA20^a^ | 0 (0.0%) | 0 (0.0%) | 0 (0.0%) | 0 (0.0%) | 0 (0.0%) |
| Total | 11 (100%) | 232 (100%) | 2 (100%) | 188 (100%) | 433 (100%) |

^a^Data is missing for these LAs as they did not deliver a HENRY programme at follow-up.

Additional Table 8. Participant age at follow-up by Local Authority

|  | Under 18 N (%) | 18-25 N (%) | 25-64 N (%) | 65+ N (%) | Prefer not to say N (%) | Missing N (%) | Total N (%) |
| --- | --- | --- | --- | --- | --- | --- | --- |
| LA |  |  |  |  |  |  |  |
| LA1 | 0 (0.0%) | 1 (2.8%) | 9 (4.4%) | 0 (0.0%) | 0 (0.0%) | 4 (2.1%) | 14 (3.2%) |
| LA2 | 0 (0.0%) | 0 (0.0%) | 5 (2.4%) | 0 (0.0%) | 0 (0.0%) | 0 (0.0%) | 5 (1.2%) |
| LA3^a^ | 0 (0.0%) | 0 (0.0%) | 0 (0.0%) | 0 (0.0%) | 0 (0.0%) | 0 (0.0%) | 0 (0.0%) |
| LA4 | 0 (0.0%) | 1 (2.8%) | 4 (1.9%) | 0 (0.0%) | 0 (0.0%) | 20 (10.6%) | 25 (5.8%) |
| LA5 | 0 (0.0%) | 8 (22.2%) | 21 (10.2%) | 1 (100.0%) | 0 (0.0%) | 28 (14.9%) | 58 (13.4%) |
| LA6 | 0 (0.0%) | 3 (8.3%) | 17 (8.3%) | 0 (0.0%) | 0 (0.0%) | 14 (7.4%) | 34 (7.9%) |
| LA7 | 0 (0.0%) | 0 (0.0%) | 0 (0.0%) | 0 (0.0%) | 0 (0.0%) | 6 (3.2%) | 6 (1.4%) |
| LA8 | 0 (0.0%) | 4 (11.1%) | 42 (20.4%) | 0 (0.0%) | 1 (50.0%) | 34 (18.1%) | 81 (18.7%) |
| LA9 | 0 (0.0%) | 2 (5.6%) | 13 (6.3%) | 0 (0.0%) | 0 (0.0%) | 11 (5.9%) | 26 (6.0%) |
| LA10 | 0 (0.0%) | 1 (2.8%) | 6 (2.9%) | 0 (0.0%) | 0 (0.0%) | 2 (1.1%) | 9 (2.1%) |
| LA11^a^ | 0 (0.0%) | 0 (0.0%) | 0 (0.0%) | 0 (0.0%) | 0 (0.0%) | 0 (0.0%) | 0 (0.0%) |
| LA12 | 0 (0.0%) | 0 (0.0%) | 17 (8.3%) | 0 (0.0%) | 1 (50.0%) | 4 (2.1%) | 22 (5.1%) |
| LA13 | 0 (0.0%) | 7 (19.4%) | 8 (3.9%) | 0 (0.0%) | 0 (0.0%) | 4 (2.1%) | 19 (4.4%) |
| LA14 | 0 (0.0%) | 1 (2.8%) | 6 (2.9%) | 0 (0.0%) | 0 (0.0%) | 4 (2.1%) | 11 (2.5%) |
| LA15 | 0 (0.0%) | 0 (0.0%) | 3 (1.5%) | 0 (0.0%) | 0 (0.0%) | 5 (2.7%) | 8 (1.8%) |
| LA16 | 0 (0.0%) | 3 (8.3%) | 18 (8.7%) | 0 (0.0%) | 0 (0.0%) | 19 (10.1%) | 40 (9.2%) |
| LA17 | 0 (0.0%) | 1 (2.8%) | 9 (4.4%) | 0 (0.0%) | 0 (0.0%) | 4 (2.1%) | 14 (3.2%) |
| LA18 | 0 (0.0%) | 3 (8.3%) | 20 (9.7%) | 0 (0.0%) | 0 (0.0%) | 20 (10.6%) | 43 (9.9%) |
| LA19 | 0 (0.0%) | 1 (2.8%) | 8 (3.9%) | 0 (0.0%) | 0 (0.0%) | 9 (4.8%) | 18 (4.2%) |
| LA20^a^ | 0 (0.0%) | 0 (0.0%) | 0 (0.0%) | 0 (0.0%) | 0 (0.0%) | 0 (0.0%) | 0 (0.0%) |
| Total | 0 (0.0%) | 36 (100%) | 206 (100%) | 1 (100%) | 2 (100%) | 188 (100%) | 433 (100%) |

^a^Data is missing for these LAs as they did not deliver a HENRY programme at follow-up.

Additional Table 9. Number and ages of children at follow-up by Local Authority

|  | Number of children^a^ | Number of children aged < 1yrs^b^ | Number of children aged 1yrs^b^ | Number of children aged 2yrs^b^ | Number of children aged 3yrs^b^ | Number of children aged 4yrs^b^ | Number of children aged 5yrs^b^ |
| --- | --- | --- | --- | --- | --- | --- | --- |
| LA |  |  |  |  |  |  |  |
| LA1 | 13 (3.7%) | 2 (2.6%) | 3 (3.8%) | 1 (1.7%) | 4 (7.0%) | 3 (7.5%) | 0 (0.0%) |
| LA2 | 6 (1.7%) | 1 (1.3%) | 3 (3.8%) | 2 (3.3%) | 0 (0.0%) | 0 (0.0%) | 0 (0.0%) |
| LA3^c^ | 0 (0.0%) | 0 (0.0%) | 0 (0.0%) | 0 (0.0%) | 0 (0.0%) | 0 (0.0%) | 0 (0.0%) |
| LA4 | 8 (2.3%) | 1 (1.3%) | 1 (1.3%) | 2 (3.3%) | 2 (3.5%) | 0 (0.0%) | 2 (5.1%) |
| LA5 | 39 (11.1%) | 11 (14.3%) | 10 (12.7%) | 5 (8.3%) | 5 (8.8%) | 4 (10.0%) | 4 (10.3%) |
| LA6 | 26 (7.4%) | 4 (5.2%) | 8 (10.1%) | 7 (11.7%) | 2 (3.5%) | 3 (7.5%) | 2 (5.1%) |
| LA7 | 0 (0.0%) | 0 (0.0%) | 0 (0.0%) | 0 (0.0%) | 0 (0.0%) | 0 (0.0%) | 0 (0.0%) |
| LA8 | 75 (21.3%) | 16 (20.8%) | 15 (19.0%) | 15 (25.0%) | 14 (24.6%) | 6 (15.0%) | 9 (23.1%) |
| LA9 | 19 (5.4%) | 7 (9.1%) | 3 (3.8%) | 0 (0.0%) | 4 (7.0%) | 1 (2.5%) | 4 (10.3%) |
| LA10 | 9 (2.6%) | 2 (2.6%) | 0 (0.0%) | 2 (3.3%) | 1 (1.8%) | 1 (2.5%) | 3 (7.7%) |
| LA11^c^ | 0 (0.0%) | 0 (0.0%) | 0 (0.0%) | 0 (0.0%) | 0 (0.0%) | 0 (0.0%) | 0 (0.0%) |
| LA12 | 24 (6.8%) | 3 (3.9%) | 7 (8.9%) | 8 (13.3%) | 4 (7.0%) | 2 (5.0%) | 0 (0.0%) |
| LA13 | 27 (7.7%) | 8 (10.4%) | 3 (3.8%) | 4 (6.7%) | 2 (3.5%) | 6 (15.0%) | 4 (10.3%) |
| LA14 | 8 (2.3%) | 4 (5.2%) | 2 (2.5%) | 1 (1.7%) | 1 (1.8%) | 0 (0.0%) | 0 (0.0%) |
| LA15 | 3 (0.9%) | 0 (0.0%) | 1 (1.3%) | 0 (0.0%) | 1 (1.8%) | 0 (0.0%) | 1 (2.6%) |
| LA16 | 28 (8.0%) | 7 (9.1%) | 6 (7.6%) | 3 (5.0%) | 2 (3.5%) | 8 (20.0%) | 2 (5.1%) |
| LA17 | 16 (4.5%) | 1 (1.3%) | 5 (6.3%) | 4 (6.7%) | 2 (3.5%) | 1 (2.5%) | 3 (7.7%) |
| LA18 | 40 (11.4%) | 7 (9.1%) | 9 (11.4%) | 5 (8.3%) | 10 (17.5%) | 4 (10.0%) | 5 (12.8%) |
| LA19 | 11 (3.1%) | 3 (3.9%) | 3 (3.8%) | 1 (1.7%) | 3 (5.3%) | 1 (2.5%) | 0 (0.0%) |
| LA20^c^ | 0 (0.0%) | 0 (0.0%) | 0 (0.0%) | 0 (0.0%) | 0 (0.0%) | 0 (0.0%) | 0 (0.0%) |
| Total | 352 (100%) | 77 (100%) | 79 (100%) | 60 (100%) | 57 (100%) | 40 (100%) | 39 (100%) |

^a^Collected as the number of children of each age per parent; data on the number of children is missing for Bexley, Doncaster, and Wirral as these LAs did not deliver a HENRY programme at follow-up.

^b^Data was missing for 188 parents.

^c^Data is missing for Bexley, Doncaster, and Wirral as these LAs did not deliver a HENRY programme at follow-up.

Additional Table 10. Participant ethnicity at follow-up by Local Authority

|  | African N (%) | Arab N (%) | Bangladeshi N (%) | Black UK N (%) | Caribbean N (%) | Chinese N (%) | English / Scottish / Welsh / Northern Irish / UK N (%) | Gypsy or Irish Traveller N (%) | Indian N (%) |
| --- | --- | --- | --- | --- | --- | --- | --- | --- | --- |
| LA |  |  |  |  |  |  |  |  |  |
| LA1 | 0 (0.0%) | 0 (0.0%) | 0 (0.0%) | 0 (0.0%) | 0 (0.0%) | 0 (0.0%) | 10 (7.4%) | 0 (0.0%) | 0 (0.0%) |
| LA2 | 0 (0.0%) | 0 (0.0%) | 0 (0.0%) | 0 (0.0%) | 0 (0.0%) | 0 (0.0%) | 3 (2.2%) | 0 (0.0%) | 0 (0.0%) |
| LA3^a^ | 0 (0.0%) | 0 (0.0%) | 0 (0.0%) | 0 (0.0%) | 0 (0.0%) | 0 (0.0%) | 0 (0.0%) | 0 (0.0%) | 0 (0.0%) |
| LA4 | 0 (0.0%) | 0 (0.0%) | 0 (0.0%) | 0 (0.0%) | 0 (0.0%) | 0 (0.0%) | 5 (3.7%) | 0 (0.0%) | 0 (0.0%) |
| LA5 | 0 (0.0%) | 0 (0.0%) | 0 (0.0%) | 0 (0.0%) | 0 (0.0%) | 0 (0.0%) | 17 (12.5%) | 0 (0.0%) | 0 (0.0%) |
| LA6 | 2 (15.4%) | 0 (0.0%) | 1 (20.0%) | 0 (0.0%) | 0 (0.0%) | 2 (100.0%) | 8 (5.9%) | 0 (0.0%) | 1 (20.0%) |
| LA7 | 0 (0.0%) | 0 (0.0%) | 0 (0.0%) | 0 (0.0%) | 0 (0.0%) | 0 (0.0%) | 0 (0.0%) | 0 (0.0%) | 0 (0.0%) |
| LA8 | 5 (38.5%) | 2 (100.0%) | 3 (60.0%) | 1 (50.0%) | 2 (50.0%) | 0 (0.0%) | 7 (5.1%) | 0 (0.0%) | 1 (20.0%) |
| LA9 | 0 (0.0%) | 0 (0.0%) | 0 (0.0%) | 0 (0.0%) | 0 (0.0%) | 0 (0.0%) | 14 (10.3%) | 0 (0.0%) | 0 (0.0%) |
| LA10 | 0 (0.0%) | 0 (0.0%) | 0 (0.0%) | 0 (0.0%) | 0 (0.0%) | 0 (0.0%) | 7 (5.1%) | 0 (0.0%) | 0 (0.0%) |
| LA11^a^ | 0 (0.0%) | 0 (0.0%) | 0 (0.0%) | 0 (0.0%) | 0 (0.0%) | 0 (0.0%) | 0 (0.0%) | 0 (0.0%) | 0 (0.0%) |
| LA12 | 5 (38.5%) | 0 (0.0%) | 0 (0.0%) | 0 (0.0%) | 2 (50.0%) | 0 (0.0%) | 1 (0.7%) | 0 (0.0%) | 0 (0.0%) |
| LA13 | 0 (0.0%) | 0 (0.0%) | 0 (0.0%) | 0 (0.0%) | 0 (0.0%) | 0 (0.0%) | 12 (8.8%) | 0 (0.0%) | 1 (20.0%) |
| LA14 | 0 (0.0%) | 0 (0.0%) | 0 (0.0%) | 0 (0.0%) | 0 (0.0%) | 0 (0.0%) | 4 (2.9%) | 0 (0.0%) | 1 (20.0%) |
| LA15 | 0 (0.0%) | 0 (0.0%) | 0 (0.0%) | 0 (0.0%) | 0 (0.0%) | 0 (0.0%) | 3 (2.2%) | 0 (0.0%) | 0 (0.0%) |
| LA16 | 1 (7.7%) | 0 (0.0%) | 1 (20.0%) | 0 (0.0%) | 0 (0.0%) | 0 (0.0%) | 10 (7.4%) | 0 (0.0%) | 1 (20.0%) |
| LA17 | 0 (0.0%) | 0 (0.0%) | 0 (0.0%) | 0 (0.0%) | 0 (0.0%) | 0 (0.0%) | 9 (6.6%) | 0 (0.0%) | 0 (0.0%) |
| LA18 | 0 (0.0%) | 0 (0.0%) | 0 (0.0%) | 0 (0.0%) | 0 (0.0%) | 0 (0.0%) | 18 (13.2%) | 0 (0.0%) | 0 (0.0%) |
| LA19 | 0 (0.0%) | 0 (0.0%) | 0 (0.0%) | 1 (50.0%) | 0 (0.0%) | 0 (0.0%) | 8 (5.9%) | 0 (0.0%) | 0 (0.0%) |
| LA20^a^ | 0 (0.0%) | 0 (0.0%) | 0 (0.0%) | 0 (0.0%) | 0 (0.0%) | 0 (0.0%) | 0 (0.0%) | 0 (0.0%) | 0 (0.0%) |
| Total | 13 (100%) | 2 (100%) | 5 (100%) | 2 (100%) | 4 (100%) | 2 (100%) | 136 (100%) | 0 (0.0%) | 5 (100%) |

^a^Data is missing for these LAs as they did not deliver a HENRY programme at follow-up.

Additional Table 10. Participant ethnicity at follow-up by Local Authority (ctd.)

|  | Irish N (%) | Mixed ethnic background N (%) | Pakistani N (%) | Any other Asian background N (%) | Any other Black / African / Caribbean background N (%) | Any other White background N (%) | Any other ethnic group N (%) | Prefer not to say N (%) | Missing N (%) | Total N (%) |
| --- | --- | --- | --- | --- | --- | --- | --- | --- | --- | --- |
| LA |  |  |  |  |  |  |  |  |  |  |
| LA1 | 0 (0.0%) | 0 (0.0%) | 0 (0.0%) | 0 (0.0%) | 0 (0.0%) | 0 (0.0%) | 0 (0.0%) | 0 (0.0%) | 4 (1.9%) | 14 (3.2%) |
| LA2 | 0 (0.0%) | 0 (0.0%) | 0 (0.0%) | 0 (0.0%) | 0 (0.0%) | 0 (0.0%) | 0 (0.0%) | 0 (0.0%) | 2 (1.0%) | 5 (1.2%) |
| LA3^a^ | 0 (0.0%) | 0 (0.0%) | 0 (0.0%) | 0 (0.0%) | 0 (0.0%) | 0 (0.0%) | 0 (0.0%) | 0 (0.0%) | 0 (0.0%) | 0 (0.0%) |
| LA4 | 0 (0.0%) | 0 (0.0%) | 0 (0.0%) | 0 (0.0%) | 0 (0.0%) | 0 (0.0%) | 0 (0.0%) | 0 (0.0%) | 20 (9.6%) | 25 (5.8%) |
| LA5 | 0 (0.0%) | 0 (0.0%) | 10 (62.5%) | 0 (0.0%) | 0 (0.0%) | 0 (0.0%) | 0 (0.0%) | 0 (0.0%) | 31 (14.8%) | 58 (13.4%) |
| LA6 | 0 (0.0%) | 2 (22.2%) | 2 (12.5%) | 0 (0.0%) | 1 (25.0%) | 0 (0.0%) | 0 (0.0%) | 0 (0.0%) | 15 (7.2%) | 34 (7.9%) |
| LA7 | 0 (0.0%) | 0 (0.0%) | 0 (0.0%) | 0 (0.0%) | 0 (0.0%) | 0 (0.0%) | 0 (0.0%) | 0 (0.0%) | 6 (2.9%) | 6 (1.4%) |
| LA8 | 1 (100.0%) | 1 (11.1%) | 1 (6.3%) | 1 (25.0%) | 2 (50.0%) | 0 (0.0%) | 9 (100.0%) | 5 (41.7%) | 40 (19.1%) | 81 (18.7%) |
| LA9 | 0 (0.0%) | 1 (11.1%) | 0 (0.0%) | 0 (0.0%) | 0 (0.0%) | 0 (0.0%) | 0 (0.0%) | 0 (0.0%) | 11 (5.3%) | 26 (6.0%) |
| LA10 | 0 (0.0%) | 0 (0.0%) | 0 (0.0%) | 0 (0.0%) | 0 (0.0%) | 0 (0.0%) | 0 (0.0%) | 0 (0.0%) | 2 (1.0%) | 9 (2.1%) |
| LA11^a^ | 0 (0.0%) | 0 (0.0%) | 0 (0.0%) | 0 (0.0%) | 0 (0.0%) | 0 (0.0%) | 0 (0.0%) | 0 (0.0%) | 0 (0.0%) | 0 (0.0%) |
| LA12 | 0 (0.0%) | 2 (22.2%) | 0 (0.0%) | 1 (25.0%) | 0 (0.0%) | 0 (0.0%) | 0 (0.0%) | 3 (25.0%) | 8 (3.8%) | 22 (5.1%) |
| LA13 | 0 (0.0%) | 0 (0.0%) | 0 (0.0%) | 1 (25.0%) | 0 (0.0%) | 0 (0.0%) | 0 (0.0%) | 1 (8.3%) | 4 (1.9%) | 19 (4.4%) |
| LA14 | 0 (0.0%) | 0 (0.0%) | 0 (0.0%) | 0 (0.0%) | 0 (0.0%) | 0 (0.0%) | 0 (0.0%) | 0 (0.0%) | 6 (2.9%) | 11 (2.5%) |
| LA15 | 0 (0.0%) | 0 (0.0%) | 0 (0.0%) | 0 (0.0%) | 0 (0.0%) | 0 (0.0%) | 0 (0.0%) | 0 (0.0%) | 5 (2.4%) | 8 (1.8%) |
| LA16 | 0 (0.0%) | 1 (11.1%) | 3 (18.8%) | 1 (25.0%) | 0 (0.0%) | 0 (0.0%) | 0 (0.0%) | 2 (16.7%) | 20 (9.6%) | 40 (9.2%) |
| LA17 | 0 (0.0%) | 1 (11.1%) | 0 (0.0%) | 0 (0.0%) | 0 (0.0%) | 0 (0.0%) | 0 (0.0%) | 0 (0.0%) | 4 (1.9%) | 14 (3.2%) |
| LA18 | 0 (0.0%) | 1 (11.1%) | 0 (0.0%) | 0 (0.0%) | 1 (25.0%) | 0 (0.0%) | 0 (0.0%) | 1 (8.3%) | 22 (10.5%) | 43 (9.9%) |
| LA19 | 0 (0.0%) | 0 (0.0%) | 0 (0.0%) | 0 (0.0%) | 0 (0.0%) | 0 (0.0%) | 0 (0.0%) | 0 (0.0%) | 9 (4.3%) | 18 (4.2%) |
| LA20^a^ | 0 (0.0%) | 0 (0.0%) | 0 (0.0%) | 0 (0.0%) | 0 (0.0%) | 0 (0.0%) | 0 (0.0%) | 0 (0.0%) | 0 (0.0%) | 0 (0.0%) |
| Total | 1 (100%) | 9 (100%) | 16 (100%) | 4 (100%) | 4 (100%) | 0 (0.0%) | 9 (100%) | 12 (100%) | 209 (100%) | 433 (100%) |

^a^Data is missing for these LAs as they did not deliver a HENRY programme at follow-up.

Additional Table 11. How did the participant hear about the HENRY programme at follow-up by Local Authority

|  | Family and friends N (%) | Leaflet N (%) | Poster N (%) | Professional N (%) | Website N (%) | Other N (%) | Missing N (%) | Total N (%) |
| --- | --- | --- | --- | --- | --- | --- | --- | --- |
| LA |  |  |  |  |  |  |  |  |
| LA1 | 1 (7.1%) | 0 (0.0%) | 0 (0.0%) | 8 (4.9%) | 0 (0.0%) | 1 (4.3%) | 4 (2.0%) | 14 (3.2%) |
| LA2 | 0 (0.0%) | 0 (0.0%) | 0 (0.0%) | 4 (2.5%) | 0 (0.0%) | 1 (4.3%) | 0 (0.0%) | 5 (1.2%) |
| LA3^a^ | 0 (0.0%) | 0 (0.0%) | 0 (0.0%) | 0 (0.0%) | 0 (0.0%) | 0 (0.0%) | 0 (0.0%) | 0 (0.0%) |
| LA4 | 0 (0.0%) | 0 (0.0%) | 0 (0.0%) | 5 (3.1%) | 0 (0.0%) | 0 (0.0%) | 20 (10.1%) | 25 (5.8%) |
| LA5 | 2 (14.3%) | 4 (16.7%) | 1 (11.1%) | 21 (12.9%) | 0 (0.0%) | 1 (4.3%) | 29 (14.6%) | 58 (13.4%) |
| LA6 | 3 (21.4%) | 4 (16.7%) | 1 (11.1%) | 9 (5.5%) | 1 (100.0%) | 2 (8.7%) | 14 (7.0%) | 34 (7.9%) |
| LA7 | 0 (0.0%) | 0 (0.0%) | 0 (0.0%) | 0 (0.0%) | 0 (0.0%) | 0 (0.0%) | 6 (3.0%) | 6 (1.4%) |
| LA8 | 1 (7.1%) | 4 (16.7%) | 1 (11.1%) | 27 (16.6%) | 0 (0.0%) | 9 (39.1%) | 39 (19.6%) | 81 (18.7%) |
| LA9 | 0 (0.0%) | 0 (0.0%) | 0 (0.0%) | 14 (8.6%) | 0 (0.0%) | 1 (4.3%) | 11 (5.5%) | 26 (6.0%) |
| LA10 | 0 (0.0%) | 2 (8.3%) | 0 (0.0%) | 2 (1.2%) | 0 (0.0%) | 2 (8.7%) | 3 (1.5%) | 9 (2.1%) |
| LA11^a^ | 0 (0.0%) | 0 (0.0%) | 0 (0.0%) | 0 (0.0%) | 0 (0.0%) | 0 (0.0%) | 0 (0.0%) | 0 (0.0%) |
| LA12 | 0 (0.0%) | 4 (16.7%) | 1 (11.1%) | 13 (8.0%) | 0 (0.0%) | 0 (0.0%) | 4 (2.0%) | 22 (5.1%) |
| LA13 | 0 (0.0%) | 0 (0.0%) | 0 (0.0%) | 15 (9.2%) | 0 (0.0%) | 0 (0.0%) | 4 (2.0%) | 19 (4.4%) |
| LA14 | 0 (0.0%) | 0 (0.0%) | 0 (0.0%) | 7 (4.3%) | 0 (0.0%) | 0 (0.0%) | 4 (2.0%) | 11 (2.5%) |
| LA15 | 1 (7.1%) | 0 (0.0%) | 0 (0.0%) | 2 (1.2%) | 0 (0.0%) | 0 (0.0%) | 5 (2.5%) | 8 (1.8%) |
| LA16 | 5 (35.7%) | 4 (16.7%) | 2 (22.2%) | 8 (4.9%) | 0 (0.0%) | 2 (8.7%) | 19 (9.5%) | 40 (9.2%) |
| LA17 | 0 (0.0%) | 0 (0.0%) | 0 (0.0%) | 5 (3.1%) | 0 (0.0%) | 1 (4.3%) | 8 (4.0%) | 14 (3.2%) |
| LA18 | 1 (7.1%) | 2 (8.3%) | 3 (33.3%) | 15 (9.2%) | 0 (0.0%) | 2 (8.7%) | 20 (10.1%) | 43 (9.9%) |
| LA19 | 0 (0.0%) | 0 (0.0%) | 0 (0.0%) | 8 (4.9%) | 0 (0.0%) | 1 (4.3%) | 9 (4.5%) | 18 (4.2%) |
| LA20^a^ | 0 (0.0%) | 0 (0.0%) | 0 (0.0%) | 0 (0.0%) | 0 (0.0%) | 0 (0.0%) | 0 (0.0%) | 0 (0.0%) |
| Total | 14 (100%) | 24 (100%) | 9 (100%) | 163 (100%) | 1 (100%) | 23 (100%) | 199 (100%) | 433 (100%) |

^a^Data is missing for these LAs as they did not deliver a HENRY programme at follow-up.

Additional Table 12. Secondary Outcomes: Child fruits and vegetables consumption (parent reported compliance) between the first and last day of the HENRY programme

|  | Pre-randomisation^a^ | | | | | | Follow-up^b^ | | | | | |
| --- | --- | --- | --- | --- | --- | --- | --- | --- | --- | --- | --- | --- |
|  | HENRY alone) (n=438) | | HENRY + Optimisation Intervention (n=443) | | Total (n=881) | | HENRY alone (n=213) | | HENRY + Optimisation Intervention (n=220) | | Total (n=433) | |
|  | % | N | % | N | % | N | % | N | % | N | % | N |
| Increase in consumption | 31.7 | 139 | 30.5 | 135 | 31.1 | 274 | 28.2 | 60 | 30.9 | 68 | 29.6 | 128 |
| Consumption remained stable | 14.4 | 63 | 13.8 | 61 | 14.1 | 124 | 20.7 | 44 | 13.2 | 29 | 16.9 | 73 |
| Decrease in consumption | 7.1 | 31 | 5.4 | 24 | 6.2 | 55 | 6.6 | 14 | 6.4 | 14 | 6.5 | 28 |
| Unknown | 46.8 | 205 | 50.3 | 223 | 48.6 | 428 | 44.6 | 95 | 49.5 | 109 | 47.1 | 204 |

^a^Calculation of outcomes used data provided for randomisation

**^b^**Calculation of outcomes used data from the most recently delivered HENRY programme during follow-up at 18 months post randomisation

Additional Table 13. Secondary Outcomes: outcome proportions and risk differences adjusted for stratification factors

|  | Unadjusted model estimates^a^ | | | | Adjusted model estimates^ab^ | | | |
| --- | --- | --- | --- | --- | --- | --- | --- | --- |
|  | N (of LAs) | Outcome (%) | RD (95% CI) | p-value | N (of LAs)^c^ | RD (95% CI) | p-value | ICC |
| Parent compliance (proportion of parents reporting an increase of 0.5 in the daily consumption of fruits and vegetables by child) |  |  |  |  |  |  |  |  |
| HENRY alone | 10 | 30.2 | -5.3 (-2.4, 13.6) | 0.563 | 10 | -7.3 (-2.5, 10.3) | 0.384 | <0.01 |
| HENRY + Optimisation Intervention | 10 | 24.9 |  |  | 10 |  |  |  |
|  |  |  |  |  |  |  |  |  |
| Proportion of centres achieving composite secondary outcome (enrolment, attendance and compliance) |  |  |  |  |  |  |  |  |
| HENRY alone | 10 | 2.0 | -5.5 (-1.4, 12.3) | 0.113 | 10 | 4.5 (-5.0, 14.1) | 0.318 | - ^f^ |
| HENRY + Optimisation Intervention | 10 | 7.5 |  |  | 10 |  |  |  |
|  |  |  |  |  |  |  |  |  |
|  |  |  |  |  |  |  |  |  |
|  |  |  |  |  |  |  |  |  |
|  |  |  |  |  |  |  |  |  |
|  |  |  |  |  |  |  |  |  |
|  |  |  |  |  |  |  |  |  |
|  |  |  |  |  |  |  |  |  |
|  |  |  |  |  |  |  |  |  |
|  |  |  |  |  |  |  |  |  |
|  |  |  |  |  |  |  |  |  |
|  |  |  |  |  |  |  |  |  |
|  |  |  |  |  |  |  |  |  |
|  |  |  |  |  |  |  |  |  |
|  |  |  |  |  |  |  |  |  |
|  |  |  |  |  |  |  |  |  |
|  |  |  |  |  |  |  |  |  |
|  |  |  |  |  |  |  |  |  |
|  |  |  |  |  |  |  |  |  |
|  |  |  |  |  |  |  |  |  |
|  |  |  |  |  |  |  |  |  |
|  |  |  |  |  |  |  |  |  |
|  |  |  |  |  |  |  |  |  |

^a^Calculation of outcomes used data from the most recently delivered HENRY programme during follow-up at 18 months post randomisation

^b^Variables controlled for in the adjusted analyses were as follows: proportion of Children’s Centres recruiting at least 8 parents per programme at randomisation, proportion of Children’s Centres retaining at least 75% of parents for a minimum of 5/8 sessions per programme at randomisation, proportion of Children’s Centres running at least one HENRY programme in 2015, size of local authority, proportion of Children’s Centres in the least / most deprived quintile as ranked by the 2015 Index of Multiple Deprivation at the Lower Layer Super Output Area. Additional variables controlled for in the adjusted analyses (with the exception of the composite secondary endpoint relating to enrolment, attendance and parent compliance) were change in the corresponding outcome from pre-programme to post-programme at pre-randomisation and the pre-programme outcome at follow-up for the most recently delivered HENRY programme.

^c^Only 16 of the 20 local authorities were included in the unadjusted analyses due to not running a programme or missing data at follow-up. The number of local authorities included in the adjusted analyses is lower for some outcomes due to missing data in the baseline covariates for the Children’s Centres that were included in the follow-up analysis for those local authorities.

Abbreviations: MD, mean difference; RD, risk difference; RR, Relative Risk; CI, confidence interval; ICC, intra-cluster correlation coefficient


Additional Table 18. Secondary outcomes: Longitudinal impact on enrolment and attendance

|  | **HENRY alone** | **HENRY + Optimisation Intervention** | **Total** |
| --- | --- | --- | --- |
| **Number of programmes delivered by centres during follow-up** | **N (%)** | **N (%)** | **N (%)** |
| 0 programmes | 39 (60.0%) | 35 (57.4%) | 74 (58.7%) |
| 1 programme | 21 (32.3%) | 19 (31.1%) | 40 (31.7%) |
| 2 programmes | 3 (4.6%) | 6 (9.8%) | 9 (7.1%) |
| 3 programmes | 2 (3.1%) | 1 (1.6%) | 3 (2.4%) |
| Total | 65 (100%) | 61 (100%) | 126 (100%) |
|  |  |  |  |
| **Number of parents recruited during follow-up programmes** | Mean (SD) | Mean (SD) | Mean (SD) |
| First programme (52 centres) | 8.4 (3.5) | 8.8 (2.8) | 8.6 (3.2) |
| Second programme (9 centres) | 8.3 (2.3) | 7.0 (1.4) | 7.4 (1.7) |
| Third programme (3 centres) | 6.0 (1.4) | 11.0 (.) | 7.7 () |
|  |  |  |  |
| **Number of weeks attended during follow-up programmes** |  |  |  |
| First programme (52 centres) | 5.1 (2.6) | 5.0 (2.5) | 5.1 (2.5) |
| Second programme (9 centres) | 3.9 (2.9) | 6.1 (2.6) | 5.3 (2.9) |
| Third programme (3 centres) | 5.7 (2.1) | 5.6 (1.7) | 5.7 (1.9) |
